# Bioactive Glycyrrhizic Acid Ionic Liquid Self‐Assembled Nanomicelles for Enhanced Transdermal Delivery of Anti‐Photoaging Signal Peptides

**DOI:** 10.1002/advs.202412581

**Published:** 2025-01-09

**Authors:** Zhuxian Wang, Jun Liu, QiuYu Chen, Yufan Wu, Yamei Li, Mingjie Ou, Shuwei Tang, Ziqing Deng, Li Liu, Cuiping Jiang, Hongxia Zhu, Qiang Liu, Bin Yang

**Affiliations:** ^1^ Dermatology Hospital Southern Medical University Guangzhou 510091 China; ^2^ School of Traditional Chinese Medicine Southern Medical University Guangzhou 510515 China

**Keywords:** computational simulations, glycyrrhizic acid, ionic liquid self‐assembled micelles, permeation enhancement, photoaging, signal peptides

## Abstract

Sigal peptides have garnered remarkable efficacy in rejuvenating photoaged skin and delaying senescence. Nevertheless, their low solubility and poor permeability bring about a formidable challenge in their transdermal delivery. To address this challenge, bioactive ionic liquids (ILs) synthesized from natural glycyrrhizic acid (GA) and oxymatrine (OMT) with eminent biocompatibility is first prepared. The components ratios and inherent forming mechanisms of GA‐OMT (GAO) are optimized by molecular dynamics simulations and density functional theory calculations. Remarkably, GAO can significantly improve the sparingly soluble properties of palmitoyl pentapeptide‐4 (PAL‐4), a model peptide drug. Subsequently, GAO self‐assembled micelles loading PAL‐4 (GAO/PAL‐4‐SM) are fabricated without additional auxiliary materials. The permeation and subcutaneous retention of PAL‐4 are significantly promoted with 10wt.% GAO‐SM. Moreover, GAO ILs facilitated PAL‐4 permeation by enhancing its miscibility and interaction with stratum corneum (SC), offering a pulling effect and micellar structures for PAL‐4, as elucidated by computational simulations. In cellular and animal photoaging experiments, GAO/PAL‐4‐SM possessed remarkable capabilities in boosting collagen and hyaluronic acid regeneration, mitigating inflammation and apoptosis, accelerating macrophage M2 polarization, thereby lessening skin wrinkles and leveraging elasticity. Collectively, the research innovatively designed an ILs self‐assembled nano‐micellar transdermal delivery system to enhance the permeability and anti‐photoaging effect of signal peptides.

## Graphical abstract

The ionic hydrogen bonding interaction between N^+^‐O^‐^ and ‐COOH predominantly drive the formation of GAO. GAO ILs can be self‐assembled into micelles with PAL‐4 (GAO/PAL‐4‐SM). GAO‐SM micelles possessed high capabilities to dissolve, load PAL‐4, and then deliver PAL‐4 to deeper skin layers. Moreover, GAO‐SM promoted the anti‐photoaging effect of PAL‐4 at the cellular and animal levels.

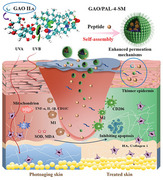

## 1 Introduction

Skin photoaging is manifested as skin wrinkles, sagging, roughness, and telangiectasia,^[^ ^1^, ^2^ ^]^ with keratinocyte vitality reduced, barrier function weakened^[^ ^3^ ^]^ as well as collagen and elastic fibers loss.^[^ ^4^ ^]^ More seriously, excessive exposure to ultraviolet (UV) radiation leads to age spots and malignant skin tumors,^[^ ^5^ ^]^ posing a substantial impact on people's welfare. Therefore, effectively managing skin photoaging and delaying senescence is the eternal pursuit of human beauty and health. The signal polypeptides are efficacious in rejuvenating photoaged skin and promoting collagen synthesis.^[^ ^6^, ^7^ ^]^ Previous literature has documented that they facilitated effective communication between skin cells. When the skin is in senescence, the signal peptides take advantage of the extracellular matrix to inform senescent cells and then send a “distress signal” to fiber cells,^[^ ^8^ ^]^ which remarkably prompts the production of collagen I and hyaluronic acid.^[^ ^9^, ^10^ ^]^ Nevertheless, the vast majority of signal polypeptides possess a low water solubility, bringing about a formidable challenge in designing and formulating cosmetic products as well as pharmaceutical preparations. Moreover, poor skin permeability was documented ascribing to its high molecular weight and strong lipophilicity, which hamper their transdermal or topical application. Palmitoyl pentapeptide‐4 (PAL‐4), a representative signal peptide synthesized by the palmitoyl lipophilic group and the hydrolyzed debris of collagen I,^[^ ^11^ ^]^ has been ubiquitously applied in various anti‐photoaging cosmetics. Unfortunately, transdermal delivery of PAL‐4 is equally challenging owing to its sparingly soluble properties, high molecular weight (802.05 Da) and log P value (3.72) (the chemical structure is provided in Figure S1 (Supporting Information).

Microneedling,^[^ ^12^ ^]^ emulsions,^[^ ^13^ ^]^ molecular modifications, and physical delivery^[^ ^14^ ^]^ are generally employed to facilitate peptide penetration. However, their application is hampered by complicated preparation procedures, skin damage, instability, and inconveniences. Transdermal permeation enhancers have garnered widespread acclaim to improve transdermal efficiency by interacting with SC lipids and keratins,^[^ ^15^, ^16^, ^17^ ^]^ and temporarily weakening their diffusing effect without causing cytotoxicity. Ionic liquids (ILs), are a class of green solvents, are organic salts consisting of organic cations and inorganic or organic anions,^[^ ^18^, ^19^ ^]^ wherein ILs derived from certain natural products possess ideal biocompatibility and biodegradability. ILs harbor high advantages of enhancing drug solubility^[^ ^20^ ^]^ and improving the transdermal absorption of drugs.^[^ ^21^, ^22^ ^]^ Especially, ILs were capable of facilitating the permeability of macromolecules, such as polypeptide compounds. It was reported that malic acid‐carnitine ILs at 5–10wt.% as a permeation enhancer significantly augmented the transdermal delivery of acetyl hexapeptide‐8, thereby improving the anti‐aging effects of the latter.^[^ ^23^ ^]^ To fulfill more effective transdermal delivery of peptides, ILs have been ingeniously designing nanomedicine formulations, such as micelles.^[^ ^24^, ^25^ ^]^ Compared to pure ILs, these transdermal delivery systems combine the benefits of both nanocarriers and “green” solvents, providing a remarkably reduced toxicity, higher penetration efficiency, and targeted and responsive delivery. Inspired by the above, IL self‐assembled micelles offer a promising avenue to increase the permeation and subcutaneous accumulation of PAL‐4, further accelerating its anti‐photoaging can be augmented.

Glycyrrhizic acid (GA), a pivotal component in the roots and rhizomes of *licorice*, is a pentacyclic triterpene saponin with remarkable anti‐inflammatory, antioxidant, and immunomodulatory effects.^[^ ^26^, ^27^ ^]^ GA treatment significantly suppressed photoaging by reducing reactive oxygen species (ROS), caspase 3, and cytochrome c levels and inhibiting hyaluronidase enzyme by blocking MMP1 activation.^[^ ^28^ ^]^ Structurally, on the one hand, three carboxyl groups with similar physicochemical characteristics co‐existed on the GA, which are good ligands to form ILs with organic cations. On the other hand, amphiphilic GA harbors a unique structure of having both hydrophilic and hydrophobic parts. We previously demonstrated that it could be self‐assembled into micelles with core‐shell nanostructure, enhancing the skin permeation of some insoluble and impermeable drugs,^[^ ^29^ ^]^ which elucidated its potential efficiency in elevating the transdermal delivery of PAL‐4. Oxymatrine (OMT) is a primary active ingredient derived from *Sophora flavescens*, which has a variety of pharmacological effects such as anti‐tumor, immunomodulation, and more importantly, anti‐aging.^[^ ^30^, ^31^ ^]^ Furthermore, OMT possessed an N^+^‐O^−^ bond and a C═O bond with strong electron‐donating capabilities on its chemical structure, which was indicative of its great potential to form ILs with GA.

Based on the above, we endeavored to design a novel GA‐OMT (GAO) self‐assembled micelles to facilitate peptide permeation, which may have the following advantages. First, no additional auxiliary materials (surfactants/cosurfactants) were required for the assembly of GAO micelles. Second, IL micelles possessed high capabilities to dissolve, load, and deliver PAL‐4. Third, bioactive GAO could synergistically enhance the anti‐photoaging effect of PAL‐4. Therefore, we hypothesized that GAO ILs self‐assembled micelles (GAO‐SM) harbored the superiority of solubilizing PAL‐4 via ILs and dramatically boosting the skin permeability of PAL‐4 through the collaborative effect of ILs and micelles. To our knowledge, no study was demonstrated to unveil the enhancing effect and mechanisms of ILs self‐assembled micelles on the skin permeability and anti‐photoaging effect of signal peptides. The IL micelles and peptides distribution in different skin layers, and their interactions with stratum corneum (SC) components were still lacking, which resulted in the uncertainty and unpredictability of its transdermal mechanisms and intrinsic style of action.

**Scheme 1:**
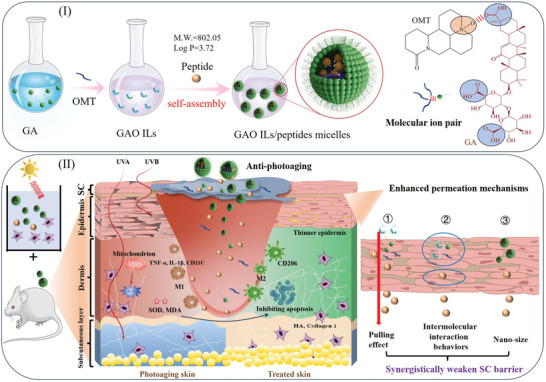
Schematic of the synthesis, permeation, and anti‐photoaging effects of GAO/PAL‐4‐SM. (I) The ionic hydrogen bonding interaction between N^+^‐O^−^ and ‐COOH predominantly drove the formation of GAO with the GA/OMT ratio of 1:3. Moreover, GAO ILs could be self‐assembled into micelles without additional auxiliary materials with PAL‐4 (GAO/PAL‐4‐SM). (II) GAO micelles possessed high capabilities to dissolve, load PAL‐4, and then deliver PAL‐4 to deeper skin layers by pulling them forward, providing a stronger interaction with skin and nano‐size. Therefore, bioactive GAO ILs promoted the anti‐photoaging effect of PAL‐4 at the cellular and animal levels.

In this comprehensive study, GAO ILs at different molar ratios were first fabricated, and Fourier transform infrared spectroscopy (FTIR), proton nuclear magnetic resonance (NMR) spectroscopy, differential scanning calorimeter (DSC), and mass chromatography were conducted to validate the successful synthesis of GAO. Next, the inherent forming mechanisms were evaluated by rheological determination, molecular dynamics simulation (MDS), and density functional theory. We also probed the cellular cytotoxicity, skin irritation, and anti‐apoptosis of GAO. Subsequently, GAO self‐assembled micelles loading PAL‐4 (GAO/PAL‐4‐SM) were novelly prepared, and their particle size, zeta potential, morphology, and critical reverse micelle concentration were exclusively characterized. On the one hand, the in vitro co‐permeation properties of PAL‐4 under the effect of different GAO‐SM were conducted on the porcine skin, which was further elaborated by computational simulations using GROMACS software. On the other hand, the inherent enhancing mechanisms of GAO‐SM were also investigated by scanning electronic microscopy (SEM), FTIR, DSC, and molecular docking. Lastly, the anti‐photoaging of GAO/PAL‐4‐SM preparations was confirmed by UVA‐induced photoaging in HSF cells as well as UV‐induced skin photoaging in C57/BL6 mice (**Scheme** **1**). Collectively, this research reveals that GAO//PAL‐4‐SM is a novel and promising strategy for enhanced transdermal delivery of impermeable peptides combining experiments and computational simulations.

## 2 Results and Discussion

### 2.1 The Synthesis and Forming Mechanisms of GAO ILs

**Figure 1:**
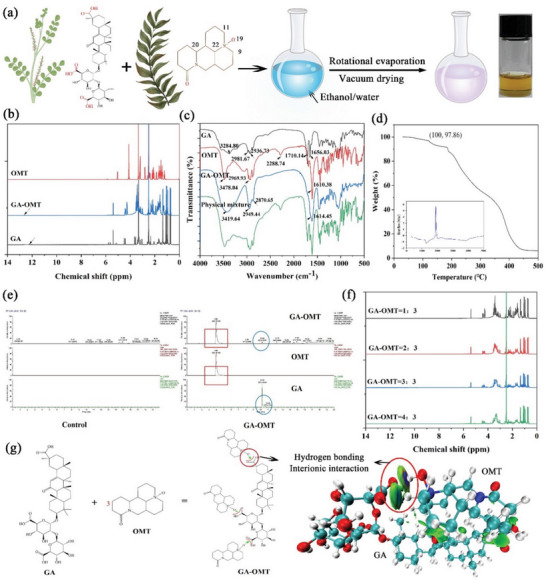
Syntheses, characterization, and the forming mechanisms of GAO ILs. a) The preparation process and the appearance of GAO (GA/OMT ratio at 1:3) b) ^1^H NMR spectra of GA, OMT, and GAO ILs; c) Infrared spectra of GA, OMT, physical mixture, and GAO ILs; d) DSC‐T_g_ thermograms of GAO; e) HPLC‐MS analysis of the GAO ILs; f) ^1^H NMR spectra of GAO with GA/OMT ratios at 1:3, 2:3, 3:3, and 4:3; g) The reaction machine of GAO.

tyStructurally, an N^+^‐O^−^ coordinate bond and a C═O bond are present on OMT (**Figure** **1**a), rendering it a high capability to accept protons in the formation of ILs.^[^ ^32^ ^]^ GA provides proton easily ascribing to the presence of three carboxylic groups with similar polar and acidity. The fabrication process of GAO is illustrated in Figure 1a. We first mixed GA and OMT at the molar ratio of 1:3. In the ^1^H NMR spectrum, upon mixing with OMT, the peak at 12–13 ppm representing the hydrogen atom for ‐COOH of GAO disappeared, and the peaks at 4–6 ppm standing for hydrogen atom attached to the ‐COOH also disappeared compared with pure GA (Figure 1b). Simultaneously, a disappearance or displacement of hydrogen atoms on the positions 9, 11, and 22 at 2.8‐3.6 ppm of OMT was observed after reacting with GA, which suggested that the hydrogen atoms attached to the N^+^‐O^−^ displayed apparent vibrations. The relative hydrogen atoms (on positions 1 and 20) attached to the C═O also disappeared after GA was mixed with OMT (Figure 1b). These demonstrated that the proton for ‐COOH had been transferred. Based on the above data, we hypothesized that a strong ionic bonding interaction and hydrogen bonding interactions were formed between the ‐COOH group for GA and the N^+^‐O^−^ or C═O site for OMT. In FTIR analysis, the peak at 2988.74 cm^−1^ representing N^+^‐O^−^ for GAO disappeared when compared with pure OMT, indicating that N^+^‐O^−^ was a vital interaction site linking with GA (Figure 1c). After mixing with OMT, the hydroxyl peak of GAO exhibited a significant blue shift from 3284.8 to 3419.64 cm^−1^ accompanied by an increased peak height compared to pure GA. These further proved the strong hydrogen bonding interaction between GA and OMT. Notably, only one characteristic peak (1614.45 cm^−1^) was detected between 1500 and 1700 cm^−1^ (Figure 1c), enunciating that only one free C═O peak existed in GAO ILs. This also testified that three carboxyl groups of GA were all involved in the interaction with N^+^‐O^−^. As a result, only the C═O group of OMT was un‐interacted and in free form in GAO ILs. Therefore, we confirmed that the ionic and hydrogen bonding interactions between N^+^‐O^−^ and ‐COOH predominantly drove the formation of GAO ILs. GA could be completely physically crosslinked with OMT to form a viscous, yellow, and clear liquid at the molar ratio of 1:3 (Figure 1a). In the DSC curve, an endothermic peak appeared when the temperature raised to ≈185 °C, indicating GAO ILs underwent a phase transition and produced thermal effects. Meanwhile, less than 2.5% mass loss for GAO was achieved at 100 °C in the T_g_ curve, demonstrating the stability of the GAO ILs (Figure 1d).

To comprehensively confirm the physical crosslinking rather than chemical interaction between GA and OMT, HPLC‐MS was carried out. It showed that no new substances were produced during the formation of ILs (Figure 1e), elucidating that the two interacted with each other predominantly through physical cross‐linking. To determine whether the ‐COOH group of GA further reacted with the C═O group of OMT to raise the intermolecular interactions of GAO ILs, we further increased the content of GA. The augment of GA content did not significantly change the position and the intensity of the C═O group (Figure 1f; Figure S2, Supporting Information). The reaction machine and interaction behaviors between GA and OMT were diagrammed in Figure 1g.

### 2.2 The Rheological Properties and Interionic Interactions of GAO

**Figure 2:**
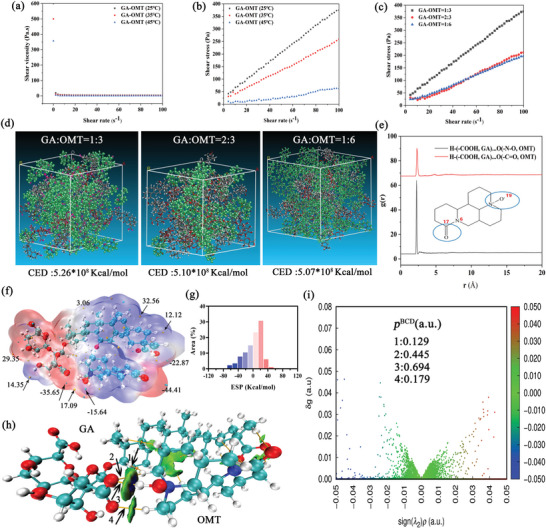
The rheological properties and interionic interaction details of GAO ILs. a) The shear viscosity and b) shear stress of GAO ILs as a function of shear rate at 25 °C, 35 °C, and 45 °C. c) Shear stress as a function of shear rate for GAO with different GA‐to‐OMT ratios at 1:3, 2:3, and 1:6; d) Snapshots and CED values of MDS for different GA‐OMT binary systems; e) RDFs of H atoms of ‐COOH in GA relative to the distance of O atoms in C═O or N^+^‐O^−^ of OMT; f, g) ESP, h) IGMH and i) AIM analysis of GAO with GA/OMT mole ratio at 1:3 in DFT analysis.

A moderate viscosity is an indispensable prerequisite^[^ ^33^ ^]^ for the topical use of GAO. Structurally, the molar ratio of the C═O group and N^+^‐O^−^ group in OMT was at 1:1. Therefore, to determine whether the ‐COOH group of GA further reacted with C═O to augment the viscosity and intermolecular strength of GAO, we doubled the content of GA. Similarly, the abundant hydroxyl group on the GA could form a high intermolecular strength with the N^+^‐O^−^ group of OMT. To prove this viewpoint, the content of GA was also doubled. As a result, GAO with GA/OMT mole ratio at 1:6 and 3:2 was selected as controls. It was observed that an increased shear rate led to a substantial reduction of shear viscosity for GAO with GA/OMT mole ratio at 1:3 (**Figure** **2**a), which was also reduced with the increasing temperature (Figure 2b). The linear relationship was well fitted between shear stress and shear rate at three kinds of temperatures (Figure 2b) and was indicative of a quintessential Newtonian fluid. Furthermore, viscosity is calculated from the slope of the linear correlation between shear stress and shear rate, which declined with the increment of temperature. Interestingly, a higher shear viscosity was achieved for GAO with GA/OMT mole ratio at 1:3 than that at 2:3 and 1:6 (Figure 2c), proving a higher intermolecular interaction.

Next, to unravel the intricacies of the interionic interactions of GAO at the molecular level, we established amorphous cell systems comprising GA and OMT at the molar ratio of 1:3, 2:3, and 1:6 using MDS analysis (Figure 2d). Amorphous cell systems could actually and thoroughly reflect the details of the interaction between different atoms in the complex GAO systems.^[^ ^34^ ^]^ We first calculated the cohesive energy density (CED) values for different binary associations, whose properties quantitatively described the strength of the interaction. A higher CED value manifested as a higher intermolecular energy.^[^ ^35^, ^36^ ^]^ We found that the CED value of GAO with GA/OMT mole ratio at 1:3 (5.26 × 10^8^ Kcal mol^−1^) was higher than that at 2:3 (5.10 × 10^8^ Kcal mol^−1^) and 1:6 (5.07 × 10^8^ Kcal mol^−1^) (Figure 2d), which suggested a stronger intermolecular entanglement and cohesion for the former, ultimately resulting a higher viscosity. As demonstrated before, the N^+^‐O^−^ group of OMT and ‐COOH group of GA held substantial implications for the formation of GAO (Figure 1). The radial distribution function (RDF) between the oxygen atom derived from N^+^‐O^−^ or C═O and the hydrogen atom from ‐COOH group in the GAO (1:3) was exclusively calculated. A higher g(r) peak also represented a stronger systematic interaction. The peaks with g(r) value < 4 Å reflected hydrogen bonding, while the peaks with g(r) value>4 Å were indicative of van der Waals force. The RDF peaks of both N^+^‐O^−^ or C═O gathered in the region < 4Å (Figure 2e), demonstrating the formation of hydrogen bonding interaction.^[^ ^26^, ^37^ ^]^ Nevertheless, the height of RDF peaks for N^+^‐O^−^‐H (‐COOH) was significantly higher than that of C═O─H (‐COOH) (Figure 2e), emphasizing the pivotal action site of N^+^‐O^−^ in OMT for ‐COOH binding, which conformed to the results of FTIR and ^1^H NMR analysis (Figure 1).

Subsequently, we discovered the density functional theory (DFT) for the ionic interactions of GAO using Gaussian software. We calculated that the concentrated electrostatic potential (ESP) of GAO was near zero, exhibiting a low range between the minimum and maximum values (Figure 2f,g), which revealed a low polarity and high structural stability.^[^ ^38^ ^]^ Next, the independent gradient model based on Hirshfeld partition (IGMH) (Figure 2h) and atoms‐in‐molecules (AIM) methods (Figure 2i), was further performed to visualize the interionic interactions.^[^ ^39^ ^]^ The large green regions between ions indicated abundant hydrogen bonding between GA and OMT. However, only a few blue dots representing the van der Waals Force were observed, which was consistent with the results of RDF analysis (Figure 2e). The orange spheres on the IGMH revealed the properties and strength of the interactions, which could be predicted from the electron density at the BCP (𝜌BCP) through a linear correlation.^[^ ^23^ ^]^ The 𝜌BCP of GAO was 0.694, 0.445, 0.179, and 0.129 (Figure 2h,i), indicating a strong hydrogen bond, which was conducive to the stability of the structure. Additionally, the IGMH iso‐surfaces and scatter maps elucidated that GAO possessed a few attractive ionic interactions (Figure 2i), enabling it suitable for permeation enhancers. The ESP, IGMH, and AIM of GAO with GA/OMT mole ratios of 1:3 and 1:6 were difficult to calculate owing to the computational limitation on the number of atoms. The mixing energy of GAO with GA/OMT mole ratio at 1:3 was −38.17 kcal mol^−1^, which was higher than that at the 2:3 (−32.22 kcal mol^−1^) and 1:6 (−31.79 kcal mol^−1^). The result was also in great accord with MDS analysis (Figure 2d). Therefore, GAO ILs with a GA/OMT mole ratio at 1:3 were selected for further analysis.

### 2.3 Biocompatibility, Conductivity, and Anti‐Apoptosis of GAO

**Figure 3:**
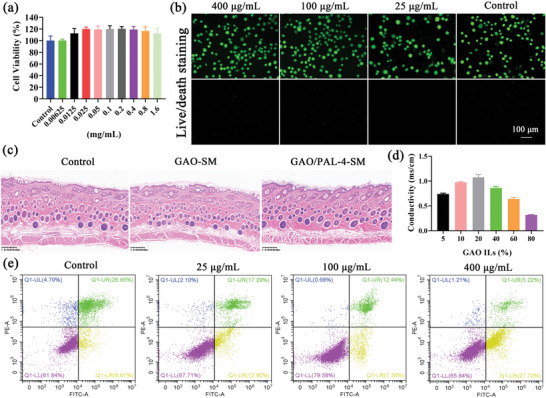
Biocompatibility, conductivity, and anti‐apoptosis of GAO ILs. a) HSF Cells viability after incubation with GAO at concentrations ranging from 6.25‐1600 µg mL^−1^ for 24 h (n = 6); b) Live‐dead staining of HSF cells treated with GAO at 25, 100, and 400 µg mL^−1^, respectively (Bar = 100 µm); c) The irritative properties of GAO‐SM and GAO/PAL‐4‐SM on the dorsal skin of guinea pig (Bar = 400 µm); d) Conductivity of GAO ILs under different water contents (n = 3); e) Apoptosis investigation of HSF cells treated with GAO at 25, 100, and 400 µg mL^−1^ using flow cytometry. Bar graphs represent mean ± SD.

To examine the biocompatibility of GAO, CCK‐8 assay and Live & Dead staining were conducted. For the CCK‐8 test, all groups exhibited high cell viability exceeding 100% (**Figure** **3**a), which indicated their excellent biocompatibility. Moreover, GAO significantly promoted cell proliferation at the concentration of 25–800 µg mL^−1^ in comparison with the control (Figure 3a). For Live & Dead staining, all groups also demonstrated remarkable cell viability, as evidenced by the brilliant green with invisible red (Figure 3b). HSF cells also demonstrated remarkable cell viability exceeding 100% when GA concentration was lower than 800 µg mL^−1^, while OMT displayed apparent cytotoxicity to HSF cells when the concentration was higher than 400 µg mL^−1^ (Figure S3a,b, Supporting Information). These suggested the cytotoxicity of OMT was significantly reduced after OMT was prepared into GAO ILs. Moreover, the histological section of guinea pig skin (Figure 3c) also showcased that the skin displayed no obvious pathological changes after treatment with GAO formulations. No necrosis, erosion, or ulceration were observed in the epidermis and dermis, without any irritating lesions such as inflammatory cell infiltration.

Generally, excessive dilution of ILs could destroy the hydrogen bond interactions between components. When GAO ILs were diluted with different amounts of water, ranging from 5%‐80%, they all could be evenly dispersed in water, presenting a transparent liquid. The electrical conductivity in water was next assessed to detect the critical point at which its structure and properties changed. The conductivity demonstrated the highest value when the GAO content of the solution was 20%, which did not decrease significantly when the GAO content was 5% or 10% (Figure 3d). However, when the content was higher than 40%, the conductivity exhibited a substantial reduction. This revealed that the GAO aqueous solution reached a critical point when the water content in the solution was lower than 80% wherein its structure and property were completely not destroyed. Furthermore, the conductivity of GA was significantly higher than OMT, indicating the predominant contributory role of GA for the conductivity of GAO (Figure S3c,d, Supporting Information).

Based on this, we evaluated the anti‐apoptosis of GAO, which is of pivotal significance for anti‐photoaging. The generation of ROS caused by UV photoaging leads to the degradation of matrix metalloproteinases‐mediated collagen, further damaging biomolecules and depleting antioxidants.^[^ ^40^ ^]^ Consequently, it impairs organelle function, especially leading to mitochondrial dysfunction, and ultimately causes inflammation and apoptosis.^[^ ^41^ ^]^ Natural aging also leads to cell apoptosis and necrosis. Thus, raising cell activities and reducing cell apoptosis plays a fundamental role in anti‐photoaging. Herein, a cell apoptosis test was conducted on HSF cells which had been cultured for 96 h in 100% convergence to simulate natural senescent cells. 4.7% of HSF cells were in a necrotic state, while 26.85% of cells gathered in the late apoptosis region in the control group (Figure 3e). Interestingly, GAO at 25 µg mL^−1^ and 100 µg mL^−1^ induced a part of late apoptotic cells to transform into normal cells and early apoptotic cells, which displayed a concentration‐effect relationship. GAO at 400 µg mL^−1^ predominantly reversed the late apoptotic cells to early apoptotic cells (Figure 3e). These results highlighted its significant potential in inhibiting apoptosis, which was beneficial for anti‐photoaging. Pure GA or OMT treatment was also capable of suppressing the cell apoptosis at 100 µg mL^−1^, but was less effective than GAO interventions, emphasizing their synergistic effect (Figure S3e, Supporting Information).

### 2.4 Self‐Assembly of GAO/PAL‐4‐SM

**Figure 4:**
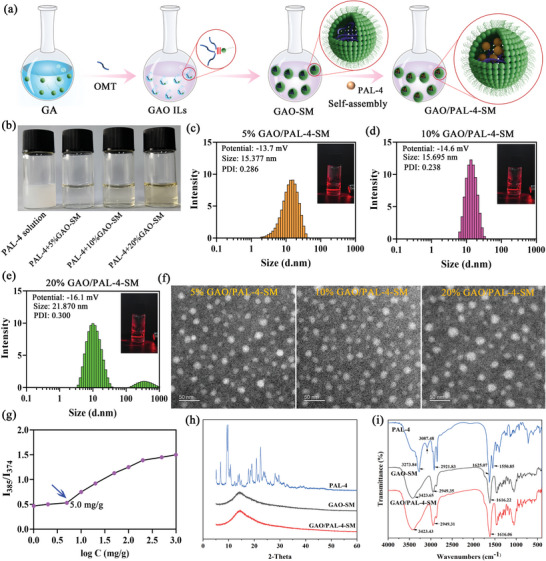
Self‐assembly of GAO/PAL‐4‐SM. a) The preparation process of GAO/PAL‐4‐SM. b) The solubility properties of PAL‐4 in water and GAO‐SM (5%, 10%, and 20%); The particle size, zeta potential, PDI, size distribution, and Tyndall light scattering effect of c) 5% GAO/PAL‐4‐SM, d) 10% GAO/PAL‐4‐SM and e) 20% GAO/PAL‐4‐SM; f) TEM images of different micelles (Bar = 50 nm); g) CMC value of 10% GAO‐SM; h) X‐ray diffraction and i) FTIR curves of PAL‐4, 10% GAO‐SM and 10%GAO/PAL‐4‐SM.

The preparation procedure of GAO/PAL‐4‐SM is diagrammed in **Figure** **4**a. To demonstrate the self‐assembly features of GAO/PAL‐4‐SM, we first evaluated the solubility of PAL‐4 in different concentrations of GAO‐SM. As observed in Figure 4b, sparingly soluble PAL‐4 (1 mg mL^−1^) was difficult to dissolve in water, and the mixture was a turbid and opaque liquid. Conversely, GAO possessed a remarkable capability of dissolving PAL‐4, exhibiting a clear yellow liquid, with a minimum solubility value of 20 mg mL^−1^ in the GAO ILs. More interestingly, GAO/PAL‐4 was still clear and transparent after diluting 5, 10, and 20 times with distilled water respectively, and contained PAL‐4 at the concentration of 1 mg mL^−1^ (Figure 4b). We hypothesized that GAO ILs were self‐assembled into nanoparticles that were capable of encapsulating large amounts of PAL‐4. Dynamic light scattering (DLS) measurements displayed that 5% GAO/PAL‐4‐SM harbored a nano‐diameter of approximately 15.377 nm and a poly‐dispersed index (PDI) value of 0.265 (Figure 4c). The nano‐size of the 10% GAO/PAL‐4‐SM exhibited no observable alternations but with a significantly lower PDI value in comparison to 5% GAO/PAL‐4‐SM. Moreover, 10% GAO/PAL‐4‐SM possessed a more uniform and concentrated distribution of the particle size, indicating more stable nano‐systems (Figure 4d). However, increasing the GAO concentration up to 20% was unable to achieve a nano‐system with higher compatibility, uniformity, and stability, but significantly enhanced their diameters and PDI values (Figure 4e). The zeta potential of the nanoparticles was −13.7 mV, −14.6 mV, and −16.1 mV when the GAO‐SM concentrations were 5%, 10%, and 20%, respectively (Figure 4c‐e). TEM examination showcased that three kinds of micelles were all spherical, whose particle sizes were similar to that of DLS (Figure 4f). A clear Tyndall light scattering effect was observed in three kinds of GAO/PAL‐4‐SM (Figure 4c‐e). Therefore, 10% GAO/PAL‐4‐SM was selected for critical micelle concentration (CMC), FTIR, and X‐ray diffraction analysis. The CMC value of 10% GAO/PAL‐4‐SM was 5 mg g^−1^, verifying the existence of a nano‐structure of micelles (Figure 4g). X‐ray diffraction analysis (Figure 4h) and FTIR (Figure 4i) together elucidated that PAL‐4 was well encapsulated in GAO‐SM, as verified by the disappearance of characteristic peaks of PAL‐4. Moreover, 10% GAO/PAL‐4‐SM exhibited higher storage stability at 0 °C and 25 °C within 3 months (Figure S4, Supporting Information).

### 2.5 The Enhancing Effect of GAO‐SM on the Permeation of PAL‐4

**Figure 5:**
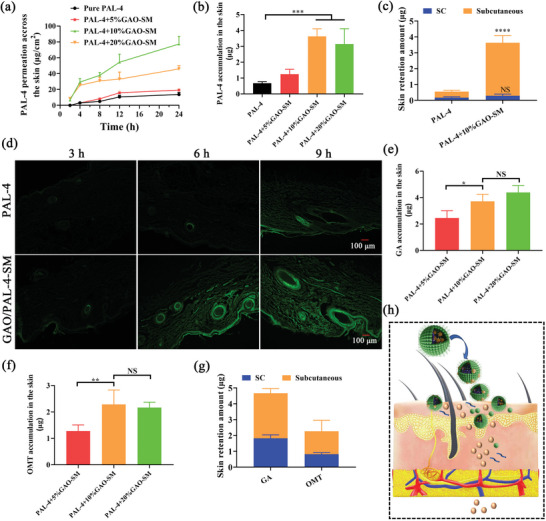
GAO‐SM remarkably enhanced the permeation and subcutaneous layers retention of PAL‐4 within 24 h. a) In vitro permeation profiles of PAL‐4 under the effect of 5%, 10%, and 20%; b) PAL‐4 accumulation in the whole skin permeation in the presence or absence of GAO‐SM; c) PAL‐4 retention in SC and subcutaneous layer with or without 10% GAO‐SM; d) The dynamic fluorescence photographs showing the FITC‐labelled PAL‐4 permeation properties with or without 10% GAO‐SM within 3, 6, and 9 h (Bar = 100 µm); e) GA and f) OMT retention from different concentration of GAO ILs‐SM in the whole skin; g) GA and OMT retention in SC and subcutaneous layer from 10% GAO‐SM. (n = 4, Data are presented as mean values ± SD, ns = not significant, *p < 0.05, **p < 0.01, ***p < 0.001, ****p < 0.001). (h) The schematic picture illustrates the enhancing permeation effect of GAO‐SM on PAL‐4.

The enhancing effect of GAO‐SM on the permeation of PAL‐4 was studied at the concentration of 5 wt.% −20 wt.% GAO‐SM. Porcine skin was used due to its similar epidermal thickness, permeability, and resistance to human skin. It displayed that 5% GAO‐SM exhibited no observable effect on the permeation of PAL‐4 (**Figure** **5**a). The cumulative permeation amount of PAL‐4 within 24 h was significantly enhanced with increasing GAO concentrations. Remarkably, a 5.64‐fold PAL‐4 permeation amount was achieved with 10% GAO‐SM compared to the control. However, PAL‐4 permeation significantly declined when GAO concentration was up to 20% (Figure 5a). In terms of PAL‐4 accumulation into the skin, a similar promoting tendency was achieved upon the treatment of GAO‐SM at different concentrations. 10% GAO displayed the highest promoting effect on PAL‐4 retention (Figure 5b). Therefore, 10% GAO‐SM was chosen to explore the effect of ILs‐SM on PAL‐4 retention in different skin layers. A 5.60‐fold PAL‐4 retention amount was obtained in the subcutaneous layers treated with 10% GAO in comparison to the control (Figure 5c). In contrast with the subcutaneous layers, a similar PAL‐4 retention amount was acquired in the SC layers of the control group and the 10% GAO‐SM group. To verify the results of the in vitro permeation study, PAL‐4 was labeled with fluorescein isothiocyanate (FITC), and the dynamic permeation of FITC‐labeled PAL‐4 with or without 10% GAO‐SM through the skin was visualized. The mean fluorescence intensity of the skin treated with GAO/PAL‐4‐SM was significantly higher than that of the control, especially after 6 h and 9 h (Figure 5d), which was consistent with the in vitro permeation investigations. Thus, 10% GAO‐SM was chosen for subsequent analysis.

Usually, the drug permeation across the skin is accompanied by the permeation of percutaneous enhancers,^[^ ^42^ ^]^ which temporarily disturbs the ordered arrangement of SC lipids or alters the secondary structure of keratin, ultimately resulting in enhanced drug permeation. Herein, both GA and OMT also accumulated in the skin (Figure 5e,f), with 45.5% permeated GA and 36.7% permeated OMT in the SC layers at the concentration of 10% GAO (Figure 5g). These played a fundamental role in the interaction between GAO and the SC components, facilitating the creation of a loosening SC barrier. Furthermore, it indicated that only a part of GAO/PAL‐4‐SM reached the epidermis and dermis during permeation. The location of GA and OMT in the epidermis and dermis was beneficial to exerting their anti‐inflammatory and antioxidant effects, exhibiting synergistic anti‐photoaging effects with PAL‐4. However, the permeation of OMT and GA in the subcutaneous layers was significantly lower than that of PAL‐4, suggesting that most of GAO/PAL‐4‐SM was broken in the skin surface or SC layers and then released PAL‐4, which was in accord with our previous study.^[^ ^29^ ^]^ Therefore, the enhanced permeation of PAL‐4 was predominantly dependent on the impact of GAO ILs on the SC layers rather than micelles. The schematic picture illustrating the enhancing permeation effect of GAO‐SM on PAL‐4 is displayed in Figure 5h.

### 2.6 The Enhancing Mechanisms of GAO‐SM on the Permeation of PAL‐4

**Figure 6:**
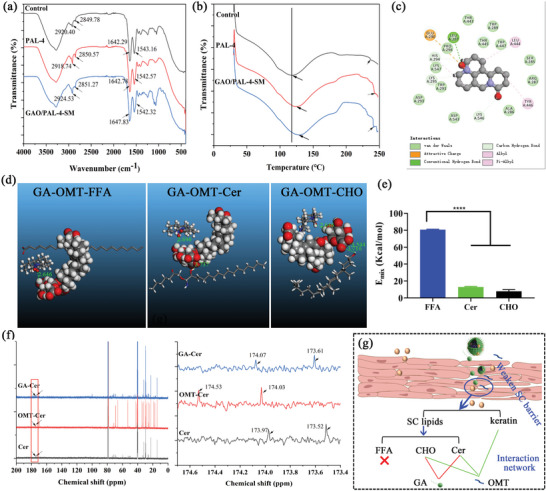
The enhancing mechanisms of GAO on the permeation of PAL‐4. a) Infrared spectra and b) DSC thermograms of the porcine skin treated with PAL‐4 or GAO/PAL‐4‐SM for 24 h; c) 2D docking associations between the residues of keratin and OMT (the black arrows stand for the N^+^‐O bond involving the formation of H‐bond with the keratin residues); d) Minimum energy ternary conformations and mixing energy of GAO binary systems with Cer, FFA or CHO; e) The mixing energy between GAO and different SC lipids. (Bar graphs represent mean ± SD, n = 4, ****p < 0.0001); f) The ^13^C NMR spectra of Cer treated with GA or OMT for 24 h, the variations of the carbonyl carbon atom of Cer in ^13^C NMR spectra analysis; g) The interaction network between different SC components and GA or OMT.

Subsequently, the permeation‐enhancing mechanisms of GAO ILs on PAL‐4 were comprehensively investigated from the viewpoint of molecular pharmaceutics. The peaks at 2920.40 cm^−1^ and 2849.78 cm^−1^ were indicative of the stretching vibration of SC lipids (V*as*CH_2_ and V*s*CH_2_), while the peaks gathered between 1650 and 1500 cm^−1^ represented the keratin peaks, including amide I and amide II (**Figure** **6**a).^[^ ^35^ ^]^ The permeation of PAL‐4 alone did not cause a significant shift of SC lipids peaks or keratin peaks, indicating a weak intermolecular force between PAL‐4 and SC components. Upon the treatment of GAO‐SM, the V*as*CH_2_ peak shifted to 2924.53 cm^−1^ in comparison to the control, accompanied by a significant blue shift of the amide I peak (Figure 6a). To collaborate on the results, DSC thermograms of different skin were recorded, which reflected the phase transition of SC components. The heat absorption peak between 115 and 125 °C was derived from the transformation of the intercellular SC lipids from the layer gel state to the liquid crystal state, whereas the absorption peak at 225–250 °C was related to the denaturation of keratin.^[^ ^29^, ^43^ ^]^ Both SC lipids peak and keratin peak moved to a higher temperature after the permeation of PAL‐4 compared to the control. The addition of GAO‐SM further remarkably facilitated the movement of SC lipids peak and keratin peak to a higher temperature (Figure 6b). These revealed that GAO weakened the SC diffusion barrier by increasing SC lipids mobility and altering keratin secondary structures.

To comprehensively assess the interaction details between GAO components and SC lipids or keratin, molecular docking was conducted using Discovery Studio and Materials Studio. First, GA, OMT, and PAL‐4 were docked to keratin (8K8H) respectively. Interestingly, only OMT possessed high compatibility with keratin (Figure 6c), whereas GA and PAL‐4 could not be well docked to keratin owing to the lack of protein ligands and their poor miscibility with keratin. The hydrogen bond and van der Waals interactions of OMT with keratin residues are in Figure 6c. OMT predominantly contacted LEU, GLU, PRO, TYR, etc., of the keratin 8K8H for binding. Remarkably, N^+^‐O^−^ was the main action site involved in the formation of the H‐bond with the keratin model. It suggested that the denaturation of keratin was predominantly caused by the OMT‐derived shell structure of GAO‐SM. Next, GAO ILs were docked with three kinds of SC lipids (Figure 6d), including ceramide II (the representative of ceramide (Cer)), tetracosanoic acid (the representative of free fatty acid (FFA)), and cholesterol (CHO). The mixing energy of different GA‐OMT‐SC lipids ternary associations were exclusively calculated, whose properties also quantitatively reflected the strength of the interaction. The closer the mixing energy to zero, the stronger the intermolecular strength. We found that Cer and CHO possessed a significantly lower E_mix_ value with GAO binary systems than FFA (Figure 6e), indicating a higher compatibility. Moreover, CHO also exhibited the highest binding energy (3.544 and 3.716 Å) with GAO, followed by Cer (3.737 Å), while FFA could not form a hydrogen bond interaction with GAO (Figure 6d). It underscored that CHO and Cer possessed a higher affinity with the GAO‐SM shell part than that of FFA. These further demonstrated that GAO mainly disturbed the steric arrangements of CHO and Cer rather than FFA in SC lipids, which increased the skin fusion for enhanced PAL‐4 permeation. Notably, CHO and Cer mainly contacted the ‐COOH group of GA for binding rather than OMT (Figure 6d).

To prove the interaction between SC lipids and GAO‐SM shell, ^13^C NMR was used to observe the chemical shift and molecular mobility of carbonyl carbon atoms in SC lipids. As demonstrated before, both Cer and CHO exhibited similar but significantly higher compatibility with GAO than FFA. Nevertheless, Cer occupied a higher amount in the SC lipids than CHO, which was usually chosen to elucidate the intermolecular interaction between SC lipids and the permeated components.^[^ ^35^, ^44^ ^]^ It was documented that the C═O group of Cer played a crucial role in linking to the permeated drugs in the SC.^[^ ^35^ ^]^ The peaks at 173.52 ppm and 173.94 represented the carbon atom of the C═O group (C_c═o_). Interestingly, both GA and OMT caused a blue shift of C_c═o_, with OMT possessing a higher capability to cause the movement of the C_c═o_ (Figure 6f). To summarize, GA and OMT synergistically caused the mobility of CHO and Cer in SC lipids, and only OMT led to the denaturation of keratin (Figure 6g), both of which jointly increased the interaction and miscibility between GAO‐SM and SC, ultimately resulting in an enhanced permeation of PAL‐4.

**Figure 7:**
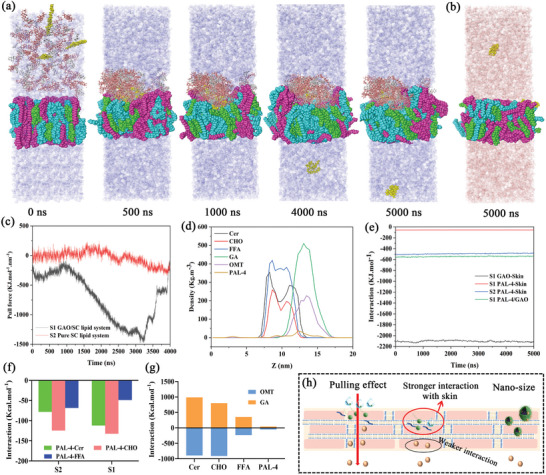
MDS analysis of PAL‐4 permeation through the SC barrier from GAO aqueous solution. The dynamic snapshots of PAL‐4 permeation across the lipid barrier in a) S1 and b) S2, with local enlargement of relevant permeation region. Blue, green, and magenta colors represent FFA, CHO, and Cer molecules, respectively; c) The pulling strength needed to pull PAL‐4 through the lipid barrier in S1 model and S2 model; d) Density distributions of FFA, Cer, CHO, GA, OMT, and PAl‐4 in the Z direction in S1 and S2; e) Total interaction force between the different components in S1 and S2; f) The interaction energy among PAL‐4 and FFA, CHO and Cer respectively; g) The interaction between GA anions or OMT cations and other components; h) GAO ILs delivered PAL‐4 to deeper skin layers by pulling them forward, providing a stronger interacting with skin and nano‐size.

Afterward, MDS simulation was performed as a persuasive tool to confirm the results of molecular docking and in vitro permeation study. A bilayer of Cer, CHO, and FFA at the molar ratio of 1:1:1 was simplified as the lipid barrier as previously demonstrated.^[^ ^45^ ^]^ As illustrated in **Figure** **7**a displaying the PAL‐4 transdermal snapshots at different times, a part of PAL‐4 permeated across the SC lipid membrane in the presence of GAO, with the rest trapped in the lipids after 5000 ns. On the contrary, all PAL‐4 molecules were accumulated in the SC lipids or kept away from the lipid interface without GAO, which was consistent with the in vitro permeation study (Figure 5). We observed that all PAL‐4 molecules are pulled to the SC lipids interface with the assistance of GA and OMT, emphasizing the pulling effect of GAO in driving PAL‐4 forward through the SC. Remarkably, GA and OMT wrapped PAL‐4 molecules into a sphere‐like mass, like self‐resembled micelles with core‐shell structure, which was in accord with the macroscopic experiment (Figure 4). These suggested that GAO facilitated PAL‐4 permeation probably by providing a strong pulling effect and nano‐size micelles for PAL‐4 (Figure 7b).

Next, we calculated the pulling strength required for PAL‐4 penetrating across the bilayer lipid barrier using the potential of mean force (PMF). PAL‐4 required a significantly lower pulling force in the GAO/SC lipid system than pure SC lipid systems in 4000 ns simulation (Figure 7c), proving that GAO helped PAL‐4 penetration through the lipid barrier. To understand the inherent enhancing mechanisms of GAO ILs, it was pivotal to gain insights into the component distribution of the system. The density distribution of Cer, CHO, and FFA along the z‐axis for GAO/lipid systems was calculated. It was illustrated that FFA possessed a more uniform distribution, whereas both CHO and Cer were accumulated in the middle of each lipid layer (Figure 7d). This suggested that CHO and Cer provided more interaction sites for GAO binding, resulting in a higher mixing energy (Figure 6d,e). In terms of ILs, both GA and OMT were more concentrated at the skin‐barrier interfaces rather than remaining in solution (Figure 7d). This further proved that GA and OMT synergistically weakened the interfacial resistance to enhance PAL‐4 permeation.

To testify to the results, the interaction between different constituents was investigated when PAL‐4 was diffused through the lipid bilayer. It showcased that PAL‐4 exhibited a significantly weaker interaction force with the lipid matrix in the GAO/SC lipid system (−54.72 kJ mol^−1^) than that in the pure SC lipid systems (−518.49 kJ mol^−1^), which was indicative of a lower diffusion resistance (Figure 7e). Moreover, the interaction between PAL‐4 and GAO was ‐563.26 kJ mol^−1^, which was significantly lower than that between GAO and the skin (−2156.2 kJ mol^−1^), revealing that the permeation‐enhancing effect of GAO was beneficial from its impact on the lipid barrier. Upon the incorporation of GAO, the interaction between PAL‐4 and Cer as well as CHO raised, while the interaction of PAL‐4 and FFA reduced (Figure 7f). Specifically, the interaction of the GA anions with other ingredients was similar to OMT cations (Figure 7g), further verifying their equally significant role in improving permeation (Figure 4g). Simultaneously, GA anions and OMT cations displayed a stronger interaction with CHO and Cer than FFA (Figure 7g), which was consistent with the molecular docking study (Figure 6d,e).

Taken together, the permeation enhancement mechanisms of GAO ILs were ascribed to the stronger interaction of GAO and lipid barrier (Cer and CHO), which remarkably weakened the interactions of PAL‐4 with SC lipids, ultimately pulling PAL‐4 molecules forward into the SC in a core‐shell micelles manner (Figure 7h).

### 2.7 GAO‐SM Impact on PAL‐4 Anti‐Photoaging Effect in Cells

**Figure 8:**
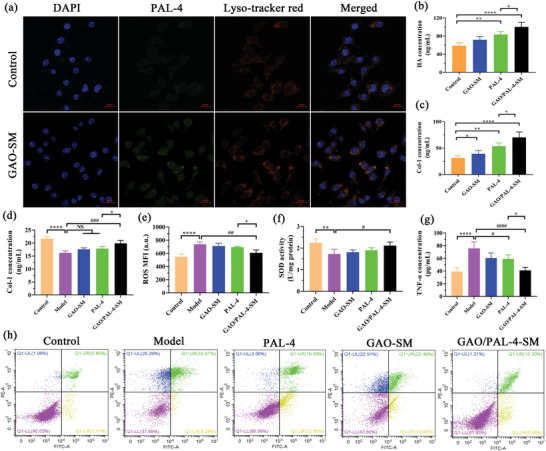
GAO‐SM increased the anti‐photoaging effect of PAL‐4 on HSF cells. a) Intracellular assimilation distribution of FTIR‐labelled PAL‐4 in HSF cells with or without 10% GAO‐SM. (blue, nucleus; green, FITC‐labelled PAL‐4; Red, lysosome; Bar = 20 µm); b) HA and c) Col‐1 intracellular levels after treatment with GAO‐SM, PAL‐4, or both, respectively; d) Col‐1 intracellular levels exposed to UV after treatment with GAO‐SM, PAL‐4, or both, respectively; e) ROS, f) SOD and g) TNF‐α levels in HSF cells exposed to UV after incubation with GAO‐SM, PAL‐4, and GAO/PAL‐4‐SM respectively; h) Apoptosis investigation of HSF cells treated with GAO‐SM, PAL‐4 and GAO/PAL‐4‐SM respectively. (n = 5, Data are presented as mean value ± SD, ^#^ *p* < 0.05, **^,##^ *p* < 0.01, ^###^ *p* < 0.01,****^,####^ *p* < 0.0001).

After unveiling the permeation‐enhancing effect and mechanisms of GAO on PAL‐4, the anti‐photoaging impact of GAO on PAL‐4 was explored using HSF cells. First, we evaluated the phagocytosis behaviors of FTIC‐labeled PAL‐4 with or without GAO‐SM. Blue meant the nucleus, while green represented the internalized PAL‐4, and red referred to the lysosome. It was obvious that GAO‐SM significantly boosted the assimilation of PAL‐4 in HSF cells, and the cellular uptake of FITC labeled PAL‐4 approximately trebled with the treatment of GAO‐SM in comparison to the control (**Figure** **8**a; Figure S5, Supporting Information). This underscored the permeation‐elevating effect of GAO‐SM at the cellular level, which laid a foundational basis for the enhanced anti‐aging effect of PAL‐4 peptides.

Hyaluronic acid (HA) plays a pivotal role in moisturizing and repairing the skin, endowing the skin elasticity, compactness, and softness.^[^ ^46^, ^47^ ^]^ Collagen is a supportive ingredient in the skin, which enhances the tension and lightens pigmentation, sagging as well as wrinkles, maintaining skin elasticity and delaying senescence.^[^ ^48^, ^49^ ^]^ Therefore, HA and collagen are the golden indicator for anti‐photoaging. Compared with the control, the incorporation of PAL‐4 significantly promoted the content of HA, which exhibited a further significant increase after the addition of GAO‐SM and PAL‐4 (Figure 8b), indicating the permeation‐promoting effect of GAO on PAL‐4. In terms of collagen I (Col‐I), a significant enhancement was observed with the treatment of pure GAO‐SM. PAL‐4 displayed a higher capacity to augment *Col‐I* content than GAO, and GAO strengthened the capability of PAL‐4 to enhance the production of Col‐I, which suggested a synergistic effect (Figure 8c). The results demonstrated that a combined application of GAO‐SM and PAL‐4 demonstrated the highest capability to raise the production of HA and Col‐I.

Next, we constructed a UV‐induced photoaged HSF cells model to evaluate the anti‐photoaging effect of GAO/PAL‐4‐SM. It showcased that the photoaged cells exhibited significantly lower HA and Col‐I levels in comparison to the control. GAO/PAL‐4‐SM demonstrated the best capacity to restore the lost HA and Col‐I content (Figure 8d; Figure S6, Supporting Information), however, no significant difference in HA levels was detected in the PAL‐4 group and GAO/PAL‐4‐SM group (Figure S6, Supporting Information). Reversely, PAL‐4 was incapable of increasing the content of Col‐I in the absence of GAO. Pure GAO was also ineffective in replenishing the HA and Col‐I in the photoaging HSF cells. Moreover, the photoaged cells displayed significantly higher ROS and TNF‐α production, with reduced superoxide dismutase (SOD) activity (Figure 8e‐g). Excessive exposure to UV contributes to leukocyte infiltration, which in turn produces hydrogen peroxide and nitric oxide accompanied by mitochondrial dysfunction, thereby resulting in an abnormal elevation of ROS.^[^ ^50^, ^51^ ^]^ The excessive production of ROS further depletes antioxidants, such as SOD and glutathione peroxide, and stimulated the production of inflammatory factors. Interestingly, GAO/PAL‐4‐SM possessed the strongest capacity to lower the content of ROS (Figure 8e) as well as TNF‐α (Figure 8g) and promoted the levels of SOD (Figure 8f). Both GAO‐SM and PAL‐4 also exhibited a remarkable decrease in the levels of TNF‐α, without an observable impact on the increased ROS and reduced SOD. These results elucidated the contributory role of GAO‐SM in strengthening the anti‐photoaging effect of PAL‐4.

Afterward, the anti‐apoptosis effects of GAO/PAL‐4‐SM on the UV‐induced HSF cells were exclusively investigated. UV irradiation caused the transformation of most normal cells to late apoptotic cells (25.29%) and necrotic cells (33.57%) in comparison to the control (Figure 8h). The addition of PAL‐4 significantly reduced the proportion of late apoptotic cells and necrotic cells, and reversed them to the late apoptotic cells or normal cells, underscoring its remarkable anti‐apoptosis efficiency. GAO‐SM was also effective in transforming the late apoptotic cells into the early apoptotic cells and normal cells, with no observable effect on the necrotic cells (Figure 8h). Consequently, a combination application of GAO‐SM and PAL‐4 demonstrated the highest efficacy in reducing the proportion of late apoptotic cells and necrotic cells, indicating a coordinated anti‐apoptosis effect. We summarized that GAO‐SM augmented the anti‐photodamaged role of PAL‐4 in promoting HA and collagen synthesis, anti‐oxidation, and anti‐inflammatory as well as inhibiting apoptosis in HSF cells.

### 2.8 GAO‐SM Impact on the Anti‐Photoaging Effect of PAL‐4 in C57/BL6 Mice

**Figure 9:**
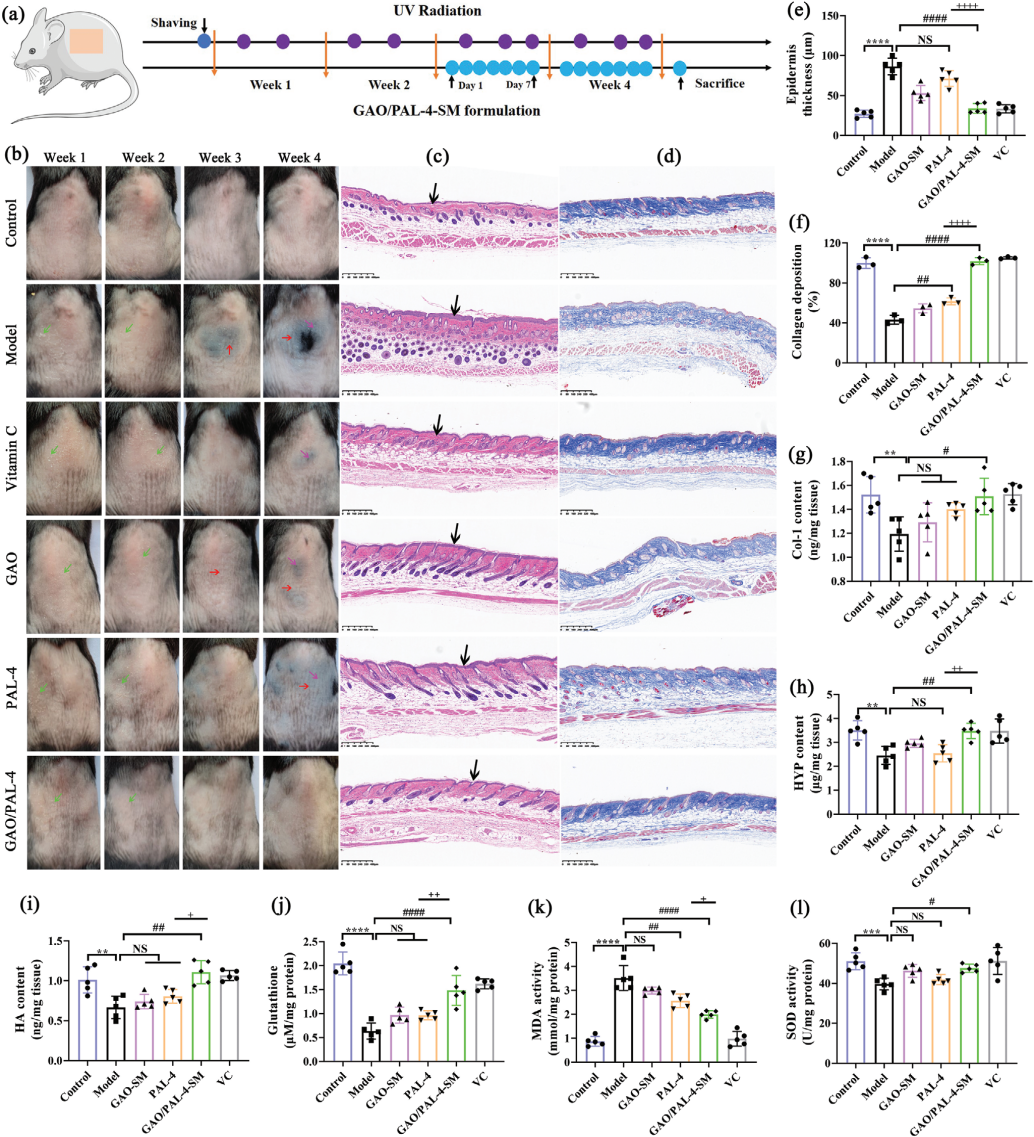
GAO strengthened the capability of PAL‐4 to lower epidermis thickness, decrease inflammation levels, supplement collagen and HA loss, and alleviate oxidative stress. a) UV irradiation and drug application timeline; b) Representative images of photoaging curation at different time points (Green arrows represent desquamation, Red arrows represent wrinkles and leathery skin, while pink arrows represent pigmentation); c) H&E and d) Masson's trichrome staining of the photoaging skin after 4 weeks (Black arrows represent epidermis, bar = 400 µm); e) Epidermal thickness calculated from HE staining; f) The relative content of collagen of different groups from Masson staining; The content of g) Col‐I, h) HYP, i) HA, j) glutathione, k) MDA and l) SOD in the skin homogenate of different groups. (Bar graphs represent mean ± SD, n = 5, **p < 0.01, ****p < 0.0001 versus control; #*p* < 0.05, ##*p* < 0.01, ##*p* < 0.0001 versus Model; ^+^ *p* < 0.05, ^++^ *p* < 0.01, ^++++^ *p* < 0.0001 versus PAL‐4; NS: no significance).

Next, we established a skin photoaging model on C57/BL6 mice using a combination of UVA and UVB irradiation to evaluate the enhancing effect of GAO‐SM on the anti‐photoaging of PAL‐4 in vivo. Compared with the control, the skin in the model group exhibited significant wrinkles, desquamation, dryness, roughness, pigmentation, and leathery appearance after 4 weeks of radiation (**Figure** **9**b; Figure S7, Supporting Information). In the PAL‐4 group, the skin still exhibited obvious desquamation, roughness, pigmentation, and leathery, signifying its incapability to reverse the photoaged skin, which can be attributed to its poor skin permeability. Conversely, with the assistance of GAO‐SM, the skin in the GAO/PAL‐4‐SM group displayed significantly fewer wrinkles and leathery, exhibiting more moisturizing, smooth, and elastic, which was similar to the normal group. These highlighted the significant potential of GAO‐SM in improving the permeability and anti‐wrinkling of PAL‐4. Furthermore, the skin wrinkles, hydration, and other disorders were improved to a certain level after being treated with pure GAO‐SM, but its efficacy was significantly poorer than that of GAO/PAL‐4‐SM and comparable to that of vitamin C. These revealed the enhanced anti‐photoaging effect of PAL‐4 after encapsulated into GAO‐SM.

To evaluate the healing effectiveness of different treatments, longitudinal sections of dorsal skin were used for HE staining (Figure 9c) and Masson's trichrome staining (Figure 9d), demonstrating the epidermis thickness, inflammatory infiltration, and collagen regeneration. We measured the dermis thickness of different groups after 2 weeks of treatments. GAO/PAL‐4‐SM demonstrated the highest efficacy in decreasing the enhanced dermis thickness than PAL‐4, and GAO‐SM also showed a similar reversing effect with PAL‐4 (Figure S8, Supporting Information). The curation of photoaging is marked by the repair of the epidermis. Epidermal thickness measurements from the HE staining elucidated that the model group exhibited the thickest epidermis skin (Figure 9e) accompanied by over‐keratinization (Figure 9b), thereby contributing to a rough and leathery skin. Moreover, a significant infiltration of inflammatory cells was observed in the dermis after 4 weeks of irradiation. The group treated with vitamin C or GAO/PAL‐4‐SM exhibited significantly thinner epidermis skin, less keratinization, and lower inflammatory infiltration, which was closer to that of normal skin tissue. Pure GAO‐SM or PAL‐4 treatment was also capable of alleviating these disorders, but was significantly less effective than GAO/PAL‐4‐SM interventions, further underscoring their synergistic effect. To quantitatively analyze the expression of inflammatory factors, we determined the level of IL‐1β in the skin using the Elisa method (Figure S9, Supporting Information). Remarkably, different groups exhibited similar tendencies with the results of HE staining, and the efficacy of GAO/PAL‐4‐SM was approaching that of vitamin C.

**Figure 10:**
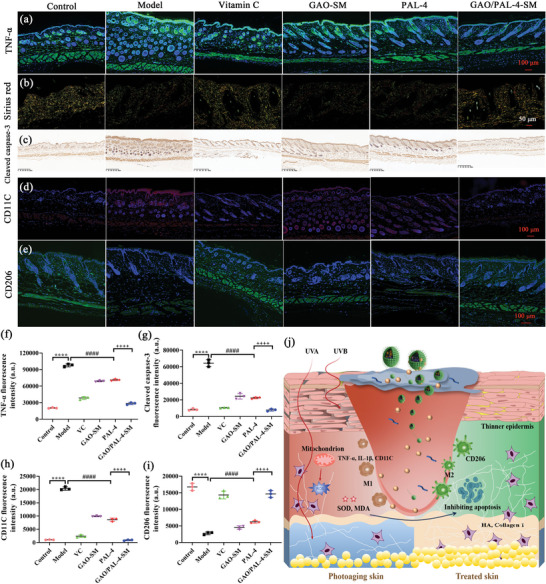
GAO augmented the anti‐photoaging effect of PAL‐4 on UV‐induced photoaging C57/BL6 mice. The immunofluorescence staining of a) TNF‐α, d) CD11c, and e) CD206 (Bar = 250 µm); b) The Sirius red staining of Col‐I and collagen III (Bar = 50 µm, the yellow to red represent Col‐I, and green represent collagen III; c) The Immunohistochemical staining of cleaved caspase‐3 (Bar = 250 µm); The mean fluorescence intensity of f) TNF‐α, g) cleaved caspase‐3, h) CD11C and i) CD206 expression assessed by Image J software. (Bar graphs represent mean ± SD, n = 3, ****p < 0.0001 versus control; ####*p* < 0.0001 versus Model; ^++++^ *p* < 0.0001 versus PAL‐4); j) The schematic picture demonstrating the molecular mechanisms of GAO‐SM enhancing the anti‐photoaging of PAL‐4.

The expression of TNF‐α in immunofluorescence examination unveiled significantly lower TNF‐α levels in the dorsal skin treated with GAO/PAL‐4‐SM in comparison to GAO‐SM and pure PAL‐4 (**Figure** **10**a,f).

Masson staining was used to analyze the content, distribution, and arrangements of the collagen in the photodamaged site. Collagen fibers primarily encompass type I and type III collagen, and type I collagen accounts for approximately 80%, which is an indispensable component to maintain skin tension and withstand tension. Type III collagen is an immature and delicate collagen fiber, which is the primary component of reticular fibers. We observed that the content of collagen fibers was significantly reduced in the model group in comparison to the control, with a chaotic arrangement and uneven distribution, thereby appearing a wrinkled and slack skin (Figure 9d,f). Upon the treatment of GAO/PAL‐4‐SM, the arrangements and content of collagen fibers were reversed to normal levels, highlighting its significant potential in ameliorating the lost collagen content. However, pure PAL‐4 and GAO‐SM treatment exhibited a significantly weaker capacity to improve collagen loss. It was documented that the content of Col‐I decreased and collagen III rose in the photoaged skin. The staining of Sirius red appearing the content of Col‐I and collagen III proved the point (Figure 10b, the enlarged picture was given in Figure S10, Supporting Information). Notably, the administration of GAO/PAL‐4‐SM significantly up‐regulated the expression of Col‐I and reduced the proportion of collagen III. In contrast, PAL‐4 was incapable of increasing the proportion of Col‐I/collagen III. We also quantitatively determined the content of Col‐I in the skin using the Elisa method, and found that GAO/PAL‐4‐SM treatment demonstrated the strongest capability to promote the content of Col‐I (Figure 9g), which was in accord with the Masson staining. HYP is a crucial and signature component of Col‐I. It was found that GAO/PAL‐4‐SM was superior to elevating the reduced HYP amount than GAO‐SM and PAL‐4 (Figure 9h), further emphasizing its superior anti‐photoaging property achieved through GAO/PAL‐4‐SM treatment. HA is another essential substance to maintain skin moisturizing, smoothness, and elasticity, as equally important as collagen. Both GAO‐SM and PAL‐4 were inefficient in refreshing the lost HA caused by UV irradiation. Conversely, GAO/PAL‐4‐SM displayed a significantly higher capacity to accelerate the production and renewal of HA than PAL‐4, which was similar to that of VC administration (Figure 9i).

To understand the mechanisms associated with skin protection against photoaging, we investigated changes in representative markers of oxidative stress in skin tissue. The model group demonstrated significantly reduced MDA levels (Figure 9j), increased SOD and glutathione levels (Figure 9k,l) when compared with the control. Remarkably, the levels of malondialdehyde (MDA), SOD, and glutathione were reversed to normal after the treatments of GAO/PAL‐4‐SM. In particular, GAO/PAL‐4‐SM possessed a higher capacity to reduce the MDA levels and enhance glutathione levels than pure PAL‐4, which was beneficial from the permeation‐enhancing effect of GAO. PAL‐4 also exhibited a significant effect in restoring the MDA level when compared to the control, with no observable impact on SOD and glutathione expression. In contrast, GAO hardly mitigated the UV‐induced accumulation of these oxidative byproducts and depletion of antioxidants (Figure 9j‐l). As demonstrated before, UV irradiation caused the apoptosis of HSF cells. Immunohistochemical test elucidated that repeated UV exposure significantly promoted the number of cleaved caspase‐3 in the epidermis, which accelerated the apoptosis of epidermal tissue (Figure 10c,g). The effect of GAO/PAL‐4‐SM on inhibiting the expression of cleaved caspase‐3 was similar to that of VC, but significantly greater than PAL‐4. Conversely, the permeation enhancer only exhibited a weak capability of lowering the expression of cleaved caspase‐3. Next, the expressions of CD11c and CD206 in the immunofluorescence staining were quantitatively evaluated to demonstrate the impact of GAO/PAL‐4‐SM on macrophage polarization. The percentage of CD11c‐positive macrophages displayed a significant reduction (Figure 10d,h) and the percentage of CD206‐positive macrophages significantly prompted (Figure 10e,i) in GAO/PAL‐4‐SM the group in comparison to the model group, which was comparable to the positive group. Conversely, both the GAO and PAL‐4 groups demonstrated a poor capacity to decrease the CD11c‐positive macrophages and enhance the percentage of CD206‐positive macrophages. These revealed the pivotal role of GAO/PAL‐4‐SM in synergistically creating an environment beneficial to M2‐mediated healing processes.

Overall, the above results underscored the imperative role of GAO in strengthening the anti‐photoaging for PAL‐4, including the reduction of epidermis thickness and inflammation level, collagen and HA supplement, oxidative stress alleviation, apoptosis inhibition as well as macrophage M2 polarization acceleration (Figure 10j).

## 3 Conclusion

In summary, our study has culminated in the development of a novel, facile, and natural GAO ILs self‐assembled micelles to augment the skin permeability and anti‐photoaging effect of signal peptide. GAO ILs with GA/OMT mole ratio at 1:3 exhibited the highest viscosity and intermolecular force, whose formation was primarily driven by the ionic bonding interaction and hydrogen bonding interaction between N^+^‐O^−^ for OMT and ‐COOH for GA. The sparingly soluble property of peptides was significantly improved under the effect of GAO ILs. Moreover, GAO ILs harbored prominent anti‐apoptosis activity and biocompatibility. Next, GAO/PAL‐4‐SM transdermal delivery systems were successfully constructed, with a diameter of ≈15 nm in a spherical shape. We found that the skin permeability and subcutaneous retention of PAL‐4 were significantly promoted upon the treatment of 10wt% GAO‐SM. Meanwhile, a part of GA and OMT was also accumulated in the SC layers, which laid a fundamental avenue for the interaction between GAO and SC components. We next gained insights into the permeation‐promotion mechanisms using computational simulations and ^1^H‐NMR. Remarkably, OMT possessed a high compatibility with keratin, while both GA and OMT had strong interaction with Cer and CHO, collaboratively increasing the miscibility with the SC. The stronger interaction of GAO and lipid barrier, the pulling effect of GAO ILs as well as the micellar structures collectively facilitated PAL‐4 molecules permeation. The cellular anti‐photoaging test indicated that GAO significantly boosted the cellular uptake of PAL‐4, contributing to a higher anti‐photoaging effect with significantly higher HA and Col‐1 levels and lower ROS, TNF‐𝛼, and SOD content. Animal experiments emphasized the potential of this multifunctional GAO/PAL‐4‐SM formulation for enhanced healing, ameliorating inflammation and apoptosis, decreasing epidermis thickness, facilitating collagen and HA regeneration, and accelerating macrophage M2 polarization in a UV radiation‐induced photoaging female mice model.

The mechanisms of GAO IL formation, facile fabrication of GAO/PAL‐4‐SM, interaction among IL enhancers, peptides, and the SC components, as well as the enhanced skin permeability and anti‐photoaging effects were explored by the comprehensive research of pharmaceutics, chemistry, materials science, biology, computer, and other disciplines. Low concentration of GAO‐SM possessed high efficiency in promoting permeability of insoluble and impermeable peptides with low side effects and cost, which improved the commercial competitiveness, endowing a good transformation prospect in cosmetic and pharmaceutical applications. Collectively, the study emphasized the development of a novel carrier for the transdermal delivery of the sparingly soluble and impermeable polypeptides, which provided method reference and theoretical support for the design of efficient IL transdermal delivery systems in various skin diseases.

## 4 Experimental Section

### Materials, Cell Lines, and Animals

OMT (purity > 98%) was supplied from Nanjing Spring & Autumn Biological Engineering Co., Ltd (Nanjing, China). Glycyrrhizic acid (purity > 98%) was from Shanghai Macklin Biochemical Co., Ltd. PAL‐4 (purity > 98%) was provided from Weihai Runhui Biotechnology Co., LTD. HPLC acetonitrile and methanol were from American supelco. Bovine serum albumin, Trypsin (0.25%), and high‐glucose Dulbecco's modified Eagle medium (DMEM‐high) were obtained from Gibco BRL Co., Ltd. CCK 8 kits were purchased from new cell and molecular biotech. Co., Ltd. Calcein‐AM/PI live/death staining kits were from Beijing Solarbio Science & Technology Co., Ltd. Annexin V‐FITC/PI double staining apoptosis detection kit was acquired from Nanjing KeyGen Biotech. Inc. SOD, MDA, Col‐I, ROS, and HA assay kits were from Nanjing Senbeijia Biotechnology Co., Ltd. IL‐1β, hydroxyproline, and glutathione knits were acquired from Enzyme‐free Biotechnology Co.Ltd. Cleaved caspase‐3 (341 034) antibody was purchased from ZEN‐BIOSCIENCE (China). CD206 (ab300621) antibody was from Abcam (America). CD11c (17342‐1‐AP) antibody was obtained from Proteintech Group (China). TNF‐𝛼 antibody (BA0131) was supplied from BOSTER (China).

The human skin fibroblast (HSF) cell lines were supplied from the central laboratory of Southern Medical University. The cells were maintained in DMEM‐high Cell Growth Medium supplemented with 10% fetal bovine serum at 37 °C, and 5% CO_2_.

Bama miniature pig was purchased from Taizhou Taihe Biotechnology Co., Ltd. Female C57/BL6 mice (6 weeks) were purchased from Gem Pharmatech Co., Ltd. All animals were housed in a room kept at 25 °C, 60% humidity and 12 h light/dark cycles and had free access to water and food. All animal studies conformed to the experiment guidelines of the Institutional Animal Care and Use at Southern Medical University.

### Synthesis of GAO ILs

The ionic liquid was based on GA with OMT at a molar ratio of 1:3. The concrete procedure was displayed as follows: 5000 mg GA was dissolved in 50 mL anhydrous ethanol/water (2:3, V/V), to which 4820 mg OMT in aqueous solution was added dropwise. The mixture was stirred at 25 °C overnight, and then the water as well as ethanol byproducts were removed using rotary evaporation at 60 °C. The resulting liquids were dried in a vacuum to obtain GAO, which was yellow, pellucid, and viscous. Successful synthesis was further testified by FTIR, proton NMR spectroscopy, DSC‐Tg, and mass chromatography. By comparison, GA‐OMT with the GA/OMT molar ratios of 2:3, 1:6, and others were also fabricated using a similar method.

### Characterization of GAO

To characterize the intermolecular interaction mode and strength in GAO ILs, FT‐IR analysis of GA, OMT, GA, and OMT physical mixture as well as GAO ILs at different ratios was carried out on Nico let iS50 FTIR spectrometer (American Thermos, New York) in the range of 400–4000 cm^−1^ with a spectral resolution of 2 cm^−1^. The ^1^H NMR data was collected on Bruker AvanceIIITM HD 600 MHz instrument (Berne, Switzerland) and then analyzed by MestReNova software. GAO was dissolved in DMSO‐d6 and then transferred into a 5 mm NMR tube for NMR measurement. The proton chemical shifts were present at δ [ppm] with tetramethylsilane as the internal standard. Moreover, DSC‐T_g_ curves of GAO were obtained on a DSC Q2000 America TA instrument under an N_2_ atmosphere (40 mL min^−1^) of a temperature range from 25 to 500 °C at a ramp rate of 10 °C min^−1^. Furthermore, mass chromatography was performed on the Orbitrap Fusion spectrometer (ThermoFisher Scientific, American) to confirm the cross‐linking details between GA and OMT. Lastly, the conductivity of the GAO ILs with different water content (w/w) was measured using a conductivity meter (INESA DDS‐307 A) under stirring conditions at 25 °C.

### Rheological Determination of GAO ILs

The viscosity of GAO ILs was evaluated by the HAAKE Viscotester iQ Air rheometer with a parallel plate‐plate geometry of 20 mm and 1 mm of gap size. The shear viscosity and shear stress of different samples were collected with the shear rate ranging from 0–120 (s^−1^) at different temperatures (25 °C, 35 °C, and 45 °C) after equilibrating for 60 s. Finally, 50 data points were recorded for each sample.

### Molecular Dynamics Simulation (MDS)

To gain insight into the forming principles and interaction behaviors of GAO ILs, MDS simulations were performed on Materials Studio software 2018. The structures of GA and OMT were generated and optimized using a forcite module under the COMPASSII force field. Subsequently, the amorphous cell module was conducted to establish systems comprising GA and OMT according to the actual proportion. The initial systems were energy optimized using the forcite module, followed by a 100 ps NVT (constant particle number, temperature, and pressure) analysis and 500 NPT (constant particle number, temperature, and volume) dynamic optimization. The NVT was equilibrated at 298 K, while the NVT ran at 305 K and 101.325 Kpa. Berendsen was used to maintain the pressure and temperature in equilibrium during the MDS process. The cohesive energy density (CED) and radial distribution function (RDF) of 500 snapshots in trajectory files were recorded, and the optimal three‐dimensional structure of GAO was downloaded.^[^ ^26^ ^]^

### The Facile Preparation and Characterization of GAO/PAL‐4‐SM

First, 5 mg, 10 mg, and 20 mg PAL‐4 were completely dissolved in 1 mL GAO under ultrasonic treatment and 65 °C, respectively. Upon the dropwise addition of water, the mixture was diluted to the solution containing 5% wt. GAO, 10% wt. GAO, and 20% wt. GAO respectively, and eventually, the concentration of PAL‐4 was at 1 mg mL^−1^. The resulting solutions were incubated at 60 °C for 2 h for the self‐assembly of GAO/PAL‐4‐SM with different concentrations of GAO.

The size distribution, zeta potential, and polymer dispersity index (PDI) of different micelles were measured by Malvern Zetasizer Nano‐ZS90 (Worcestershire, Britain). The CMC of GAO/PAL‐4‐SM was determined by pyrene‐labeled methods as we previously revealed.^[^ ^29^ ^]^ The morphology of micelles was photographed by transmission electron microscopy (TEM, H‐7650) at a voltage of 60 kV. The successful encapsulation of PAL‐4 in GAO‐SM was confirmed by FTIR and X‐ray diffraction.

### Co‐Permeation of PAL‐4 and GAO In Vitro

The in vitro experiments were performed using TP‐6 Franz diffusion cells (Tianjin Jingtuo Instrument Technology Co., Ltd, China) on fresh and intact porcine skin. The porcine skin devoid of subcutaneous tissue was positioned between the receptor compartment and the donor cells, with the dermis facing downwards. The receiver cell was filled with diethylene glycol ethyl ether/phosphate‐buffered saline (PBS, pH 7.4) (2:3, V/V), kept at 32 °C, and stirred at 350 rpm. After that, 0.3 mL GAO/PAL‐4‐SM containing 5% GAO, 10% GAO, or 20% GAO (wt.%) was applied on the porcine skin, respectively. PAL‐4 solution was regarded as control. Samples of 1 mL were collected at regular intervals (2, 4, 6, 8, 10, 12, and 24 h) and supplemented with the same prewarm receptor medium. The content of PAL‐4 at different time points was determined by high‐performance liquid chromatography (HPLC, Agilent 1260, USA) equipped with a C18 column (5 µm, 4.6 × 250 mm) and a DAD detector. The detailed HPLC method for PAL‐4, GA, and OMT was given in Method S1 (Supporting Information).

After the transdermal experiments, the skin pieces were withdrawn and washed with normal saline solutions. The SC layers and subcutaneous layers were separated using the tape stripping method, cut into small pieces, and treated by ultrasound extraction. The PAL‐4, GA, and OMT retention amounts in the SC and subcutaneous layers were determined by HPLC, respectively. Additionally, the fluorescence photographs of FITC‐labeled PAL‐4 through the skin at 3 h, 6 h, and 9 h were observed by a laser confocal microscope (CLSM 800, ZEISS, Germany).

### The Interaction among PAL‐4, GAO, and the Skin

*FTIR and DSC*: FTIR and DSC were together to elucidate the displacements of lipid and keratin peaks in the SC, further unveiling the inherent permeation‐enhancing mechanisms of GAO. The treated skin sample was obtained as the in vitro permeation test, cleaned, and used for FTIR and DSC as the aforementioned.

*Molecular Docking*: Molecular docking was performed to demonstrate the potential interactions between keratins or lipids and GA, OMT, or PAL‐4 using Discovery Studio and Materials Studio 2018. First, the structures of GA, OMT, and PAL‐4 were optimized under the CHARMm force field in The Clean Geometry and Full Minimization modules using Discovery Studio. Simultaneously, the structure of keratin (4ZRY) was downloaded from the Human Protein Data Bank. The poly‐configuration of keratin protein was removed with incomplete amino acid residues added and hydrogenated. GA, OMT, and PAL‐4 were donated as ligand molecules, while keratin was seen as receptor molecules using the receptor‐ligand interactions module and input Typed Protein Molecule module. The simulations were conducted in the Receptor‐Ligand Interaction Module and the Input Protein Molecule Module. Binding energies and docking scores were calculated after obtaining the optimal 2D and 3D conformations of the receptor‐ligands.

Afterward, the interactions among GA, OMT, and different SC lipids compounds were elucidated by Materials Studio software. Ceramide II and tetracosanoic acid were regarded as the representative ceramide (Cer) and free fatty acid (FFA). The minimum energy of GAO binary associations was first obtained using the blend module and compass II force. Subsequently, GAO served as the Base, while Cer, FFA, and cholesterol (CHO) were regarded as the screen. The interaction parameters of GAO‐SC lipids ternary systems were calculated by the blends module.

*Molecular Dynamics Analysis*: The molecular dynamics simulations of PAL‐4 through the SC membrane under the effect of GAO were performed on GROMACS software. A bilayer of Cer, FFA, and CHO at a molar ratio of 1:1:1 was considered as the skin barrier.^[^ ^23^, ^45^ ^]^ Next, the topological parameters for PAL‐4, Cer, CHO, FFA, GA, and OMT were generated using CHARMM36m. The atomic coordinates of these molecules were placed in a cubic box, and solvated with the tip3p water model, and the PAL‐4/skin associations were constructed with pure water and 10 wt.% GAO using the Membrane Builder module in CHARMM‐GUI. In the energy minimization step, the unreasonable intramolecular contacts were removed by conjugate gradient algorithm to converge the maximum force lower than 100 kJ mol^−1^ nm^−1^. Subsequently, the simulations were carried out in the NPT systematic with a pressure of 1.0 bar, an isotropic coupling coefficient of 4.5 × 10^−5,^ and a coupling constant of 12.0 ps using the Parrinello‐Rahman algorithm. Under the V‐rescale algorithm, the temperature was maintained at 298.15 K with a coupling constant of 1.0 ps. Additionally, the short‐range interaction cutoff distance was set to 1.0 nm, and the remote interactions were calculated using the Particle Mesh Ewald algorithm. Finally, a run of 10 s constrained simulation of each system with a time step of 2 fs to observe the transdermal process of PAL‐4 and calculate its tensile force.

*Skin Irritation Test*: The skin irritation of GAO and GAO/PAL‐4‐SM was assessed on the dorsal skin of the guinea pig (Method S2, Supporting Information).

### Cell Experiments

*Cell Cytotoxicity*: The cell cytotoxicity of GAO ranging from 25–1600 µg mL^−1^ was evaluated on HSF cells using CCK‐8 and Live and death staining methods (Method S3, Supporting Information).

*Cellular Uptake*: HSF cells were plated in the glass‐bottom dish with a density of 2 × 10^3^ cells per well and grown for 24 h, respectively. Subsequently, the cells were treated with FTIC‐labelled PAL‐4 or GAO/FTIC‐labelled PAL‐4‐SM (20 µg mL^−1^) for 2 h. After assimilation, the lysosome was stained with Lyso‐Tracker Red for another 30 min. After washing with cold PBS, the cells were located with paraformaldehyde solution (4%, w/v) for 10 min. Finally, the cell nucleus was stained with DAPI (1 µg mL^−1^) and then observed and captured using CLSM (CLSM 800, ZEISS, Germany).

*Anti‐Apoptosis*: HSF cells were seeded in 12‐well plates at a density of 2 × 10^5^ cells per well and grown to 100% coverage for 96 h. Different concentrations of GAO were incorporated respectively, which were further cultured for another 48 h. After rinsing with cold PBS for three times, the cells were stained with AnnexinV‐FITC and Propidium iodide, followed by detection by a flow cytometer (BECKMAN COULTER, CytoFLEX, USA).

To evaluate the anti‐apoptosis induced by UV, the cells were pre‐treated with GAO, PAL‐4, and GAO/PAL‐4‐SM for 24 h respectively, while PBS served as a control. The cells were irradiated by UVA at a radiation energy of 12.5 J cm^−2^ and then detected by flow cytometer as demonstrated above.

*Anti‐Photoaging*: HSF cells were seeded in a 12‐well plate at a density of 1 × 10^5^ cell per well and treated with GAO, PAL‐4, and GAO/PAL‐4‐SM for 48 h, respectively. HA and Col‐I expressions in the cell supernatant were measured using Elisa methods based on the manufacturer's instructions. Meanwhile, a photoaging model was established by UVA irradiation at a radiation energy of 12.5 J cm^−2^ on HSF cells. The HA, Col‐I, ROS, TNF‐α, and SOD contents in the supernatant were determined after 24 h pre‐treatment with different samples according to the manufacturer's protocols. Moreover, the cells were stained with AnnexinV‐FITC and Propidium iodide. After rinsing with cold PBS for three times, followed by detection by a flow cytometer (BECKMAN COULTER, CytoFLEX, USA).

*Skin Anti‐Photoaging Test on the C57/BL6 Mice*: Six‐week‐old female C57/BL6 mice were used for skin anti‐photoaging test. After one week of acclimatization, the back of the mice was depilated and adapted for 24 h. Six separate groups were set: control, photoaging model (UV), vitamin C as a positive control, GAO‐SM (UV+GAO‐SM), PAL‐4 (UV+PAL‐4), and GAO/PAL‐4‐SM (UV+GAO/PAL‐4‐SM), with five in each group. The air‐napped mice were placed under a preheated UV chamber for 20 cm, and a fan was used to reduce the temperature of the skin surface during irradiation. The UV radiation cycle was maintained for 4 weeks. A dose of MED (minimal erythema dose) of combinations of 1200 mJ cm^−2^ for UVA and 200 mJ cm^−2^ for UVB was given during the first and second weeks, while two MED doses were used in the third and fourth weeks. In the last two weeks, GAO‐SM, PAL‐4, GAO/PAL‐4‐SM, and VC were applied on the skin once a day, respectively. The control group was treated with PBS. The photoaging procedure, drug application, and healing timeline are illustrated in Figure 9a. Photographs were taken at fixed intervals to observe and record the desquamation, wrinkles, leathery appearance, edema, and pigmentation on the skin. At the end of the experiments, the treated skin was collected for HE, Masson, immunohistochemistry (cleaved caspase‐3), Sirius red and immunofluorescence (TNF‐α, CD86 and CG206) staining, as well as Elisa assay for superoxide dismutase (SOD), hyaluronic acid (HA), collagen 1 (Col‐I), glutathione, hydroxyproline (HA), malondialdehyde (MDA) and IL‐1β. The epidermis thickness, total collagen content, the expression of TNF‐α, cleaved caspase‐3, CD11C, and CD206 were quantitatively determined by ImageJ software.

### Statistical Data Analysis

Quantitative data were expressed as mean ± standard deviation (SD). The statistical data processing was performed using GraphPad Prism 8 or Origin 2021. One‐way analysis of variance or two‐way ANOVA with Dunnett's multiple comparisons was used to analyze the significance of the difference. *P* < 0.05 was regarded as statistically significant.

## Conflict of Interest

The authors declare no conflict of interest.

## Supporting information



Supporting Information

## Data Availability

The data that support the findings of this study are available from the corresponding author upon reasonable request.
