# Supplementary material for: Bioactive Glycyrrhizic Acid Ionic Liquid Self‐Assembled Nanomicelles for Enhanced Transdermal Delivery of Anti‐Photoaging Signal Peptides
Source: Adv Sci (Weinh). 2025 Jan 9;12(8):2412581. doi: 10.1002/advs.202412581 (PMC11848569; doi:10.1002/advs.202412581)
Supplement: Supplementary file 1 — Supporting Information [file ADVS-12-2412581-s001.docx]

Supplementary materials

Bioactive glycyrrhizic acid ionic liquid self-assembled nanomicelles for enhanced transdermal delivery of anti-photoaging signal peptides

*Zhuxian Wang^1^, Jun Liu^1^, QiuYu Chen^1^, Yufan Wu^2^, Yamei Li^2^, Mingjie Ou^2^, Shuwei Tang^2^, Ziqing Deng^2^, Li Liu^2^, Cuiping Jiang*^2^, *Hongxia Zhu*^2^, *Qiang Liu^1,^* ****,*** *Bin Yang^1,^* *******

^1^Dermatology Hospital, Southern Medical University, Guangzhou, China

^2^School of Traditional Chinese Medicine, Southern Medical University, Guangzhou, China.

*Corresponding author.

Dermatology Hospital, Southern Medical University, Guangzhou 510091, China (Bin Yang, Qiang Liu)

Email address: yangbin1@smu.edu.cn (Bin Yang); liuqiang@smu.edu.cn (Qiang Liu)

**Method S1**

For PAL-4, the mobile phase comprised combinations of A (acetonitrile), B (0.1% Trifluoroacetic acid in water, v/v) with an elution gradient as follows: 0 min, 60% A; 20 min, 30% A; 25 min, 60% A; UV detection wavelength was set to 215 nm. For OMT, the mobile phase consisted of acetonitrile and 0.1% phosphoric acid aqueous solution (7:93, v/v) with a flow rate of 1 mL/min. The detection wavelength was set at 215 nm. For GA, the mobile phase consisted of combinations of A (acetonitrile), B (methanol) and C (0.1% phosphate in water, v/v) at a flow rate of 1.0 mL/min with an elution gradient as follows: 0 min, 15% A and 75% C; 5 min, 22% A and 66% C; 10 min, 32% A and 54% C; 20 min, 45% A and 35% C; 20–30 min, 45% A and 35% C; 40 min, 15% A and 10% C.

**Method S2**

The dorsal hair of guinea pig was carefully shaved and depilated with an area of 2.5 cm×2.5 cm, which was used for subsequent analysis after 3 day of acclimatization. 0.5 g GAO-SM, GAO/PAL-4-SM was applied to the skin, and then covered with gauze and removed after 4 h. PBS was served as control. Skin reactions were observed and photographed at 1, 24, 48 and 72 h after removal of the formulations. At the end of the experiment, the skin was fixed with 4% paraformaldehyde, dehydrated, embedded, deparaffinized and stained with hematoxylin-eosin. Lastly, the skin sections were photographed using a digital slice scanner (Kf-pro-020, KFBIO, China).

**Method S3**

HSF cells (5×10^5^ cells/well) in good growth condition were plated in 6-well plates and cultured overnight, followed by incubated with GAO ranging in 1600-25 µg/mL for 24 h. After that, 10 µLCCK-8 reagent was added and incubated with cells for 2 h. A microplate reader (MULTISKAN FC, Thermo scientific, USA) was then used to measure the optical absorbance at 450 nm.

Additionally, cells incubated with GAO at different concentrations were stained with calcein-AM for 20 min, and PI dyes 5 min respectively according to the manufacturer’s protocols. After staining, the cell morphology was captured under a fluorescence microscope (DMi8, Leica, Germany).


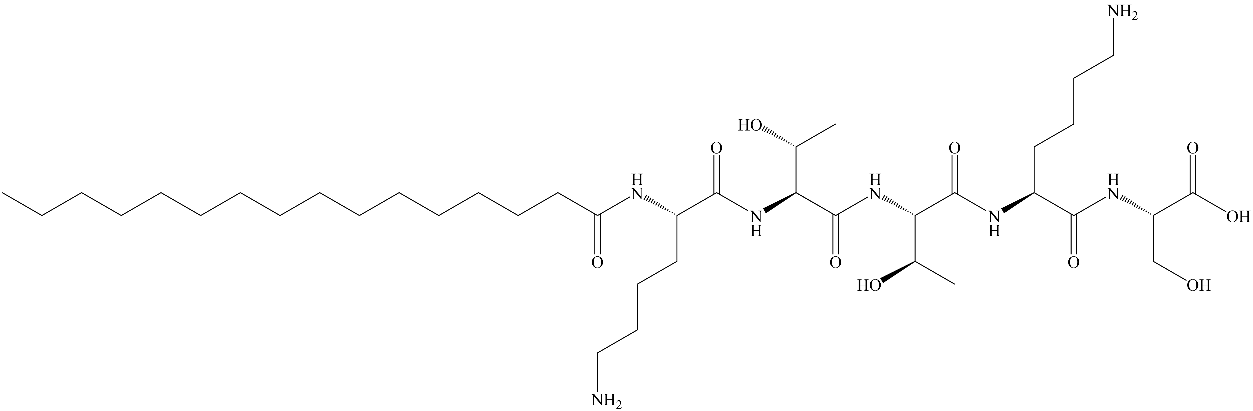


**Figure S1** The chemical structure of PAL-4


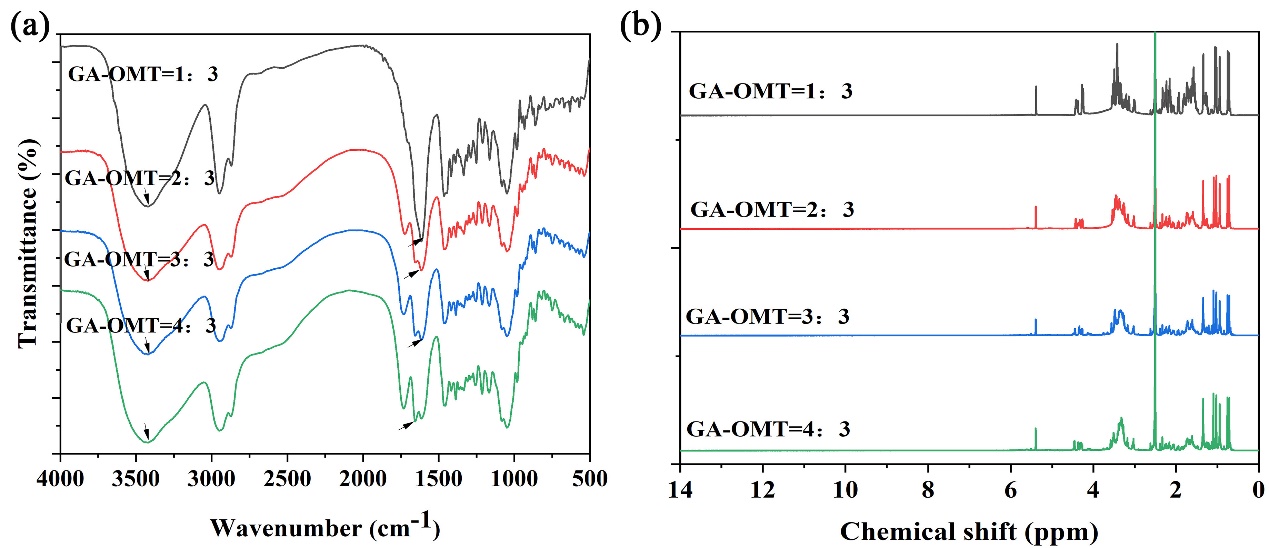


**Figure S2** Infrared spectra of GAO ILs with GA/OMT ratios of 1:3, 2:3, 3:3 and 4:3.


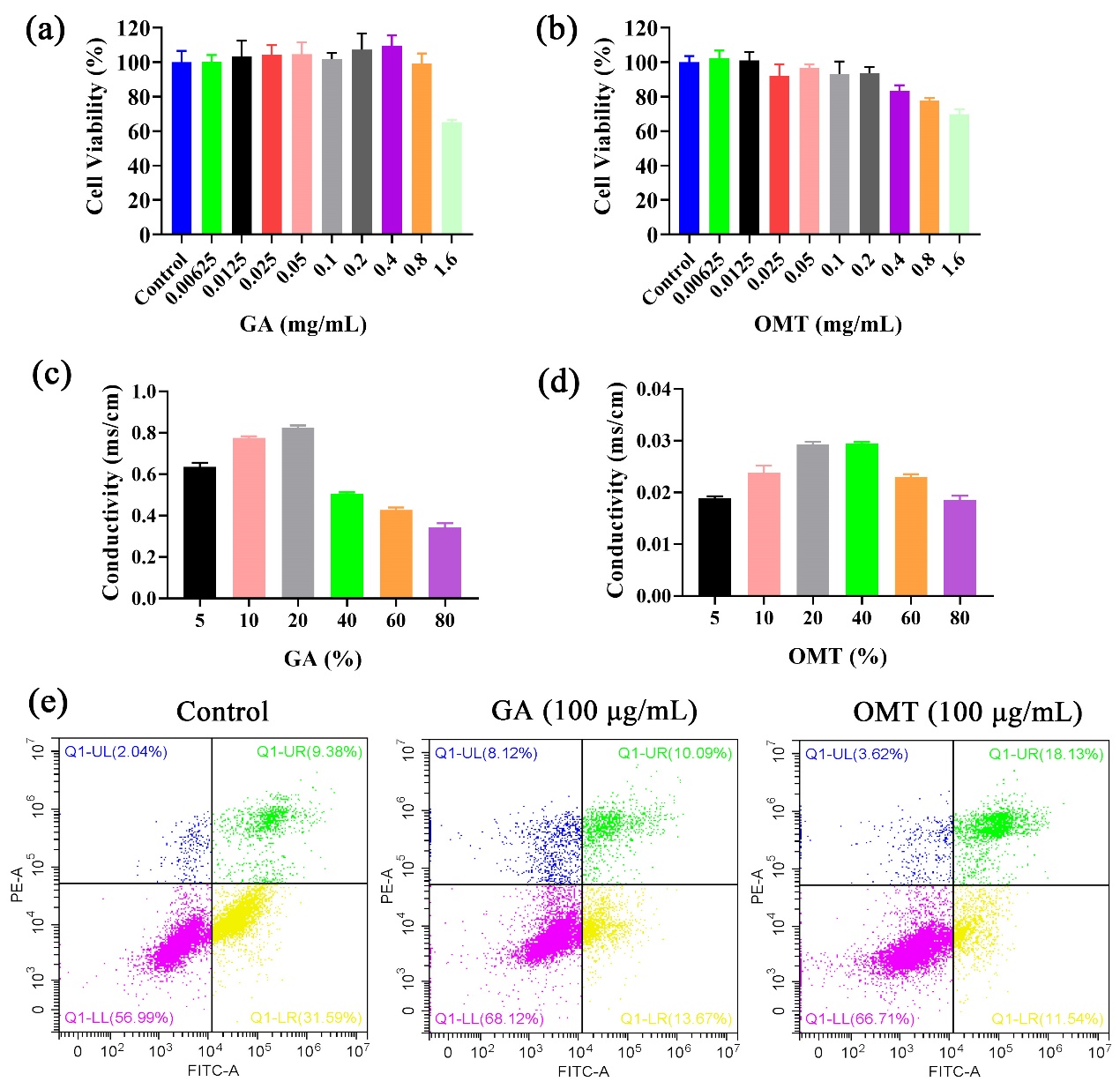


**Figure S3** The biocompatibility, conductivity and anti-apoptosis assays for GA and OMT. (a) HSF Cells viability after incubation with (a) GA or (b) OMT at the concentration ranging from 6.25-1600 μg/mL for 24 h; Conductivity of (c) GA or (d) OMT under different water contents (n=3); (e) Apoptosis investigation of HSF cells treated with GA or OMT at 100 μg/mL using flow cytometry.


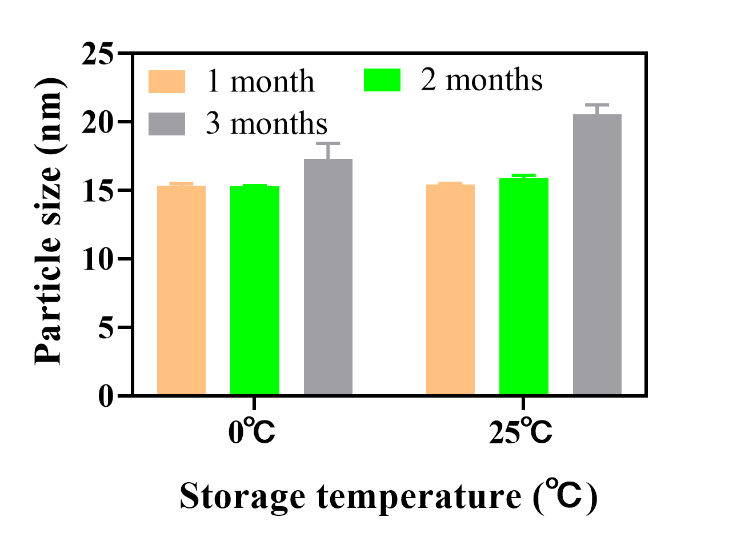


**Figure S4** The particle size of 10% GAO/PAL-4-SM at 0℃ and 25℃ for different storage time （n=3）


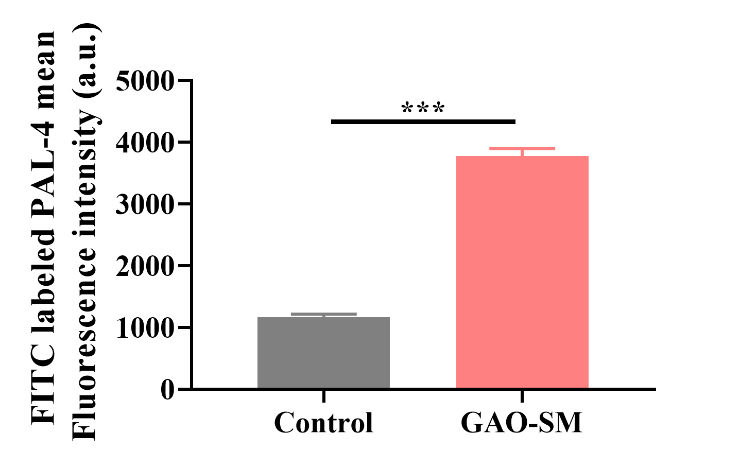


**Figure S5** The fluorescence intensity of FITC labeled PAL-4 in HSF cells with or without 10% GAO-SM (n=3, Data are presented as mean value±SD, ***p < 0.001).


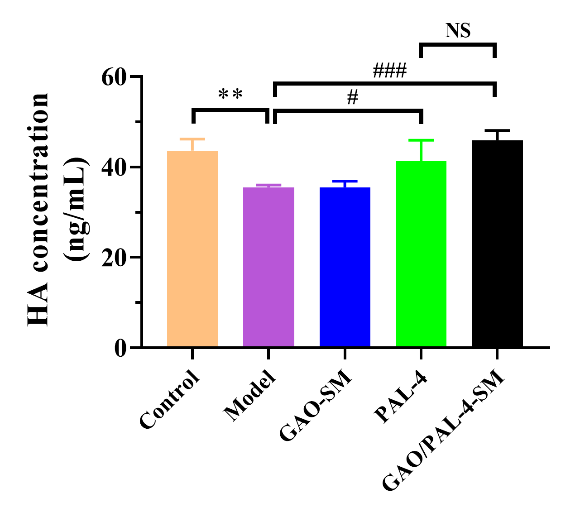


**Figure S6** HA levels in HSF cells exposed to UV after treatment with GAO-SM, PAL-4 or the both, respectively. (n=4, Data are presented as mean value±SD, #*p*< 0.05, ***p*< 0.01, ###*p*< 0.001).


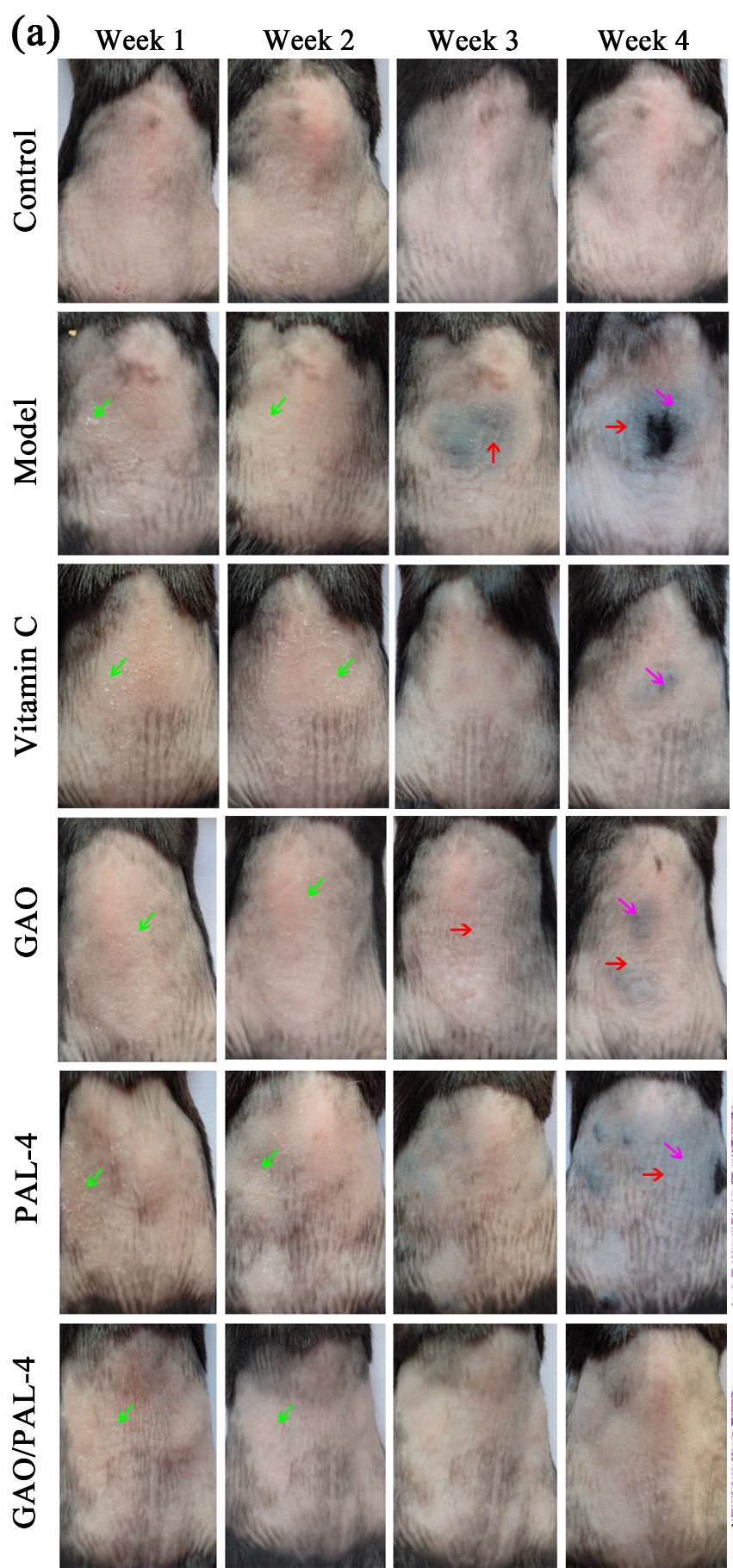


**Figure S7** Representative images of photoaging curation at different time points (Green arrows represent desquamation, Red arrows represent wrinkles and leathery skin, while pink arrows represent pigmentation)


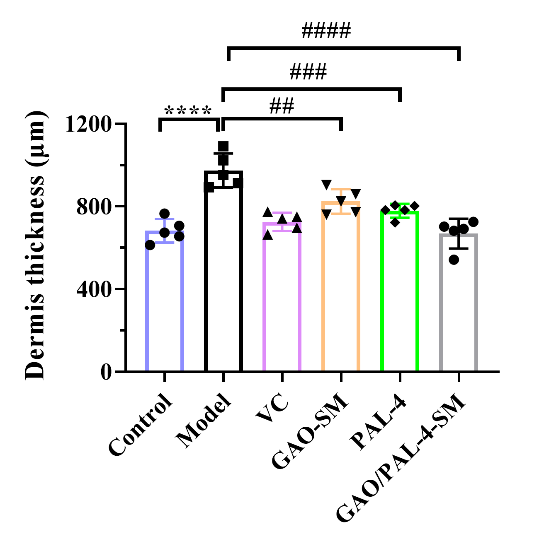


**Figure S8** Dermal thickness calculated from HE staining after different treatments for photoaging. (n=5, Data are presented as mean value±SD, ****p<0.0001 versus control; ##p<0.01, ###p<0.001, ####p< 0.0001 versus Model)


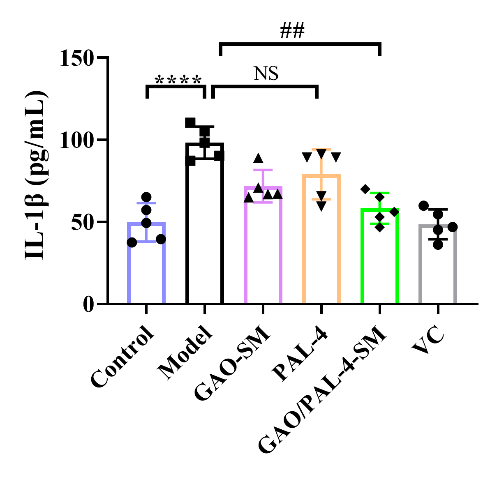


**Figure S9** The content of IL-1β in the skin homogenate of different groups. (Bar graphs represent mean±SD，n=5, *****p* < 0.0001 versus control; ##*p*< 0.01 versus PAL-4)


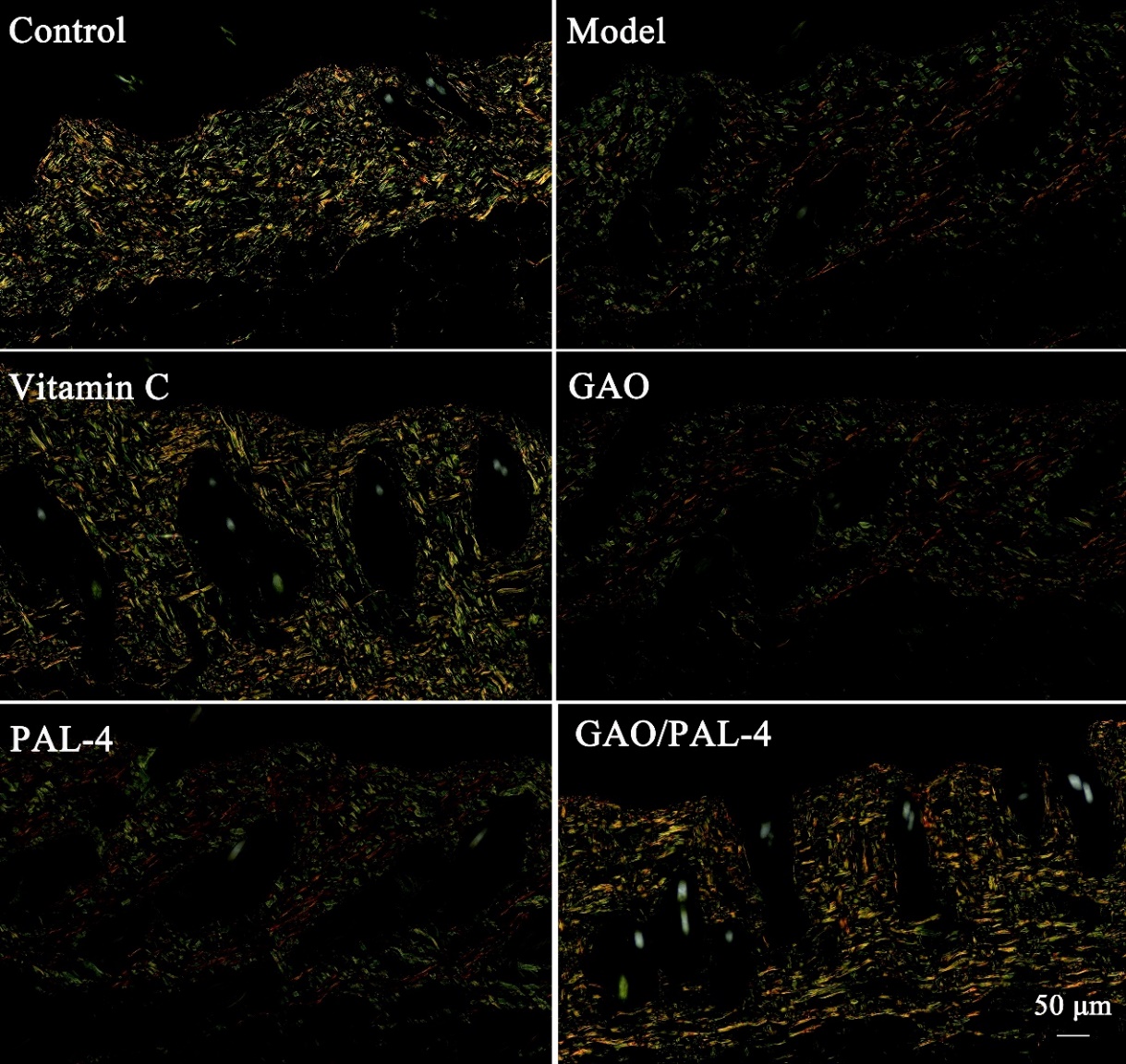


**Figure S10** The sirius red staining of collagen I and collagen III (Bar=50 μm, the yellow to red represent collagen I, and green represent collagen III
